# Genetic exchanges are more frequent in bacteria encoding capsules

**DOI:** 10.1371/journal.pgen.1007862

**Published:** 2018-12-21

**Authors:** Olaya Rendueles, Jorge A. Moura de Sousa, Aude Bernheim, Marie Touchon, Eduardo P. C. Rocha

**Affiliations:** 1 Microbial Evolutionary Genomics, Institut Pasteur, Paris, France; 2 UMR 3525, CNRS, Paris, France; University of Warwick, UNITED KINGDOM

## Abstract

Capsules allow bacteria to colonize novel environments, to withstand numerous stresses, and to resist antibiotics. Yet, even though genetic exchanges with other cells should be adaptive under such circumstances, it has been suggested that capsules lower the rates of homologous recombination and horizontal gene transfer. We analysed over one hundred pan-genomes and thousands of bacterial genomes for the evidence of an association between genetic exchanges (or lack thereof) and the presence of a capsule system. We found that bacteria encoding capsules have larger pan-genomes, higher rates of horizontal gene transfer, and higher rates of homologous recombination in their core genomes. Accordingly, genomes encoding capsules have more plasmids, conjugative elements, transposases, prophages, and integrons. Furthermore, capsular loci are frequent in plasmids, and can be found in prophages. These results are valid for Bacteria, independently of their ability to be naturally transformable. Since we have shown previously that capsules are commonly present in nosocomial pathogens, we analysed their co-occurrence with antibiotic resistance genes. Genomes encoding capsules have more antibiotic resistance genes, especially those encoding efflux pumps, and they constitute the majority of the most worrisome nosocomial bacteria. We conclude that bacteria with capsule systems are more genetically diverse and have fast-evolving gene repertoires, which may further contribute to their success in colonizing novel niches such as humans under antibiotic therapy.

## Author summary

Previous works showed that bacteria encoding capsules are better colonizers and are dominant in most environments suggesting a positive role for capsules in the genetic diversification of bacteria. Yet, it has been repeatedly suggested, based almost exclusively studies in few model species, that such bacteria are less diverse and engage in fewer genetic exchanges. Here, we reverse the current paradigm and show that bacteria encoding capsules have larger and more diverse gene repertoires, which change faster by horizontal gene transfer and recombination. Our study alters the traditional view of the capsule as a barrier to gene flow and raises novel questions about the role of capsules in bacterial adaptation.

## Introduction

Extracellular capsules constitute the outermost layer of cells. They can be synthesized through different genetic pathways [1, 2] and although some capsule types can be of proteic nature, notably the poly-γ-d-glutamate or PGA capsules produced by *Bacillus anthracis* [3], the vast majority are high molecular weight polysaccharides made up of repeat units of oligosaccharides. Most polysaccharidic capsule loci are highly variable and encode numerous polymer-specific enzymes, which determine the oligosaccharidic combination of the capsule (*i*.*e*. its serotype). Such diversity is generated by horizontal gene transfer and recombination across species but also within species [4–6].

Capsules are best known for their role in clinical settings, where they increase survival upon phagocytosis by eukaryotic cells [7, 8] and lower the sensitivity to antibiotics [9, 10]. They are thus considered a major virulence factor. However, capsules also play a critical role in the environment because they protect the cells from physical and chemical stresses. For example, they increase survival under desiccation and protect from antimicrobial peptides [10–13]. They also enhance bacterial survival rates in mixed species communities and complex environments by, for instance, protecting bacteria from bacteriocins [12–15]. Furthermore, capsules can prevent other bacteria from invading a niche by diminishing the ability of competitors to attach to a surface or to integrate an existing biofilm [15, 16]. Our previous study revealed that capsules are encoded in half of the bacterial genomes across all major phyla [17]. They are more frequent in environmental bacteria than in pathogens, being almost completely absent in obligatory pathogens. Additionally, species encoding capsules colonize a larger range of environments [17].

It has been often proposed that capsules hinder the transfer of genetic information between cells, presumably because they constitute a physical barrier to DNA acquisition. This was documented *in vitro* [18–21], *in vivo* [22] and using computational analyses [23], but mainly in one single naturally transformable species (*Streptococcus pneumoniae*). It has been shown that one phylogenetic cluster of *S*. *pneumoniae* strains lacking capsular loci is a reservoir of genetic diversity for the whole species and these strains recombine at higher rates than the capsulated strains [23]. However, a recent study in the same species reported a positive correlation between capsule thickness and recombination rate [24]. Indeed, capsules can provide a competitive advantage by favouring colonization and withstanding harsh environments, *e*.*g*., tolerating higher concentrations of antibiotics. These stressful conditions are also those that favour high rates of genetic exchange, since the latter accelerate adaptation. Hence, one would expect a positive association between the presence of capsules and the rates of homologous recombination (HR), that spread favourable alleles in populations, and of horizontal gene transfer (HGT), that drive the acquisition of novel genes. Nonetheless, the role of capsules in transduction and conjugation is ambiguous. While capsules protect bacteria from being infected by some phages [25–28], other phages require the presence of capsular polysaccharides to attach, and subsequently infect, bacterial cells [29, 30]. It is unclear if DNA conjugation is affected at all by the presence of a capsule. Early reports indicate that encapsulated *Haemophilus influenzae* are efficient donors and recipients of conjugative plasmids, and suggest that conjugation is more effective between cells sharing the same capsular serotype than across serotypes [31].

Whilst the effect of capsules in shaping the frequency of genetic exchanges remains controversial, several studies have shown that HGT [4, 32] and HR [5, 33, 34] drive the rapid evolution of bacterial capsules. Hence, the effect of capsules in restricting transfer affects their own rates of genetic diversification. To clarify the role of capsules in bacterial adaptation, and in their own evolution, it is thus essential to understand whether they affect genetic exchanges. For this, we inferred the rates of HR and HGT in 127 species across the prokaryote phylogeny. We then characterized the presence of capsules, mobile genetic elements (MGEs), and bacterial defence systems in over 5000 complete genomes. The integration of these results revealed that, contrary to the current paradigm, there are strong positive associations between the presence of capsular loci and genetic exchange.

## Results

### Species encoding capsules exchange more genes

We sought to test whether bacterial species encoding capsule systems (C_sp_+) have different rates of genetic exchange compared to the others (C_sp_-). To do so, we searched for capsule systems in the genomes of 137 species with more than four complete genomes publicly available. Among these, 122 bacterial species—62 Proteobacteria, 31 Firmicutes, 11 Actinobacteria, eight Tenericutes, four Chlamydiae, three Bacteroidetes two Spirochaetes and one Thermotogae—and five archaea encoded a capsule in more than 80% of the strains (C_sp_+) or in less than 20% (C_sp_-) (S1 Dataset, see Methods). We tried to use the ten remaining species to assess if capsule acquisition was followed by increases or decreases of genetic exchanges. In these few species, capsulated strains were usually in a single monophyletic clade, precluding the detection of significant statistical signal. This shows that the presence of a capsule locus is stable even if capsules serotypes change rapidly. Naturally, the locus may not always be expressed.

Among the remaining 127 species, 68 were C_sp_+ (54%) (S1 Fig), which is a frequency close to that of the database of complete genomes (57%, see Methods). The number of genomes per species was similar within the group of C_sp_+ and C_sp_- (*P* = 0.93, Wilcoxon test). C_sp_+ were also evenly split between naturally transformable and other species (*P* = 0.74, χ^2^ test, S2A Fig). On the other hand, the average size of the genomes of C_sp_+ is larger than that of C_sp_- (Wilcoxon test, *P* = 0.0001).

**Fig 1:**
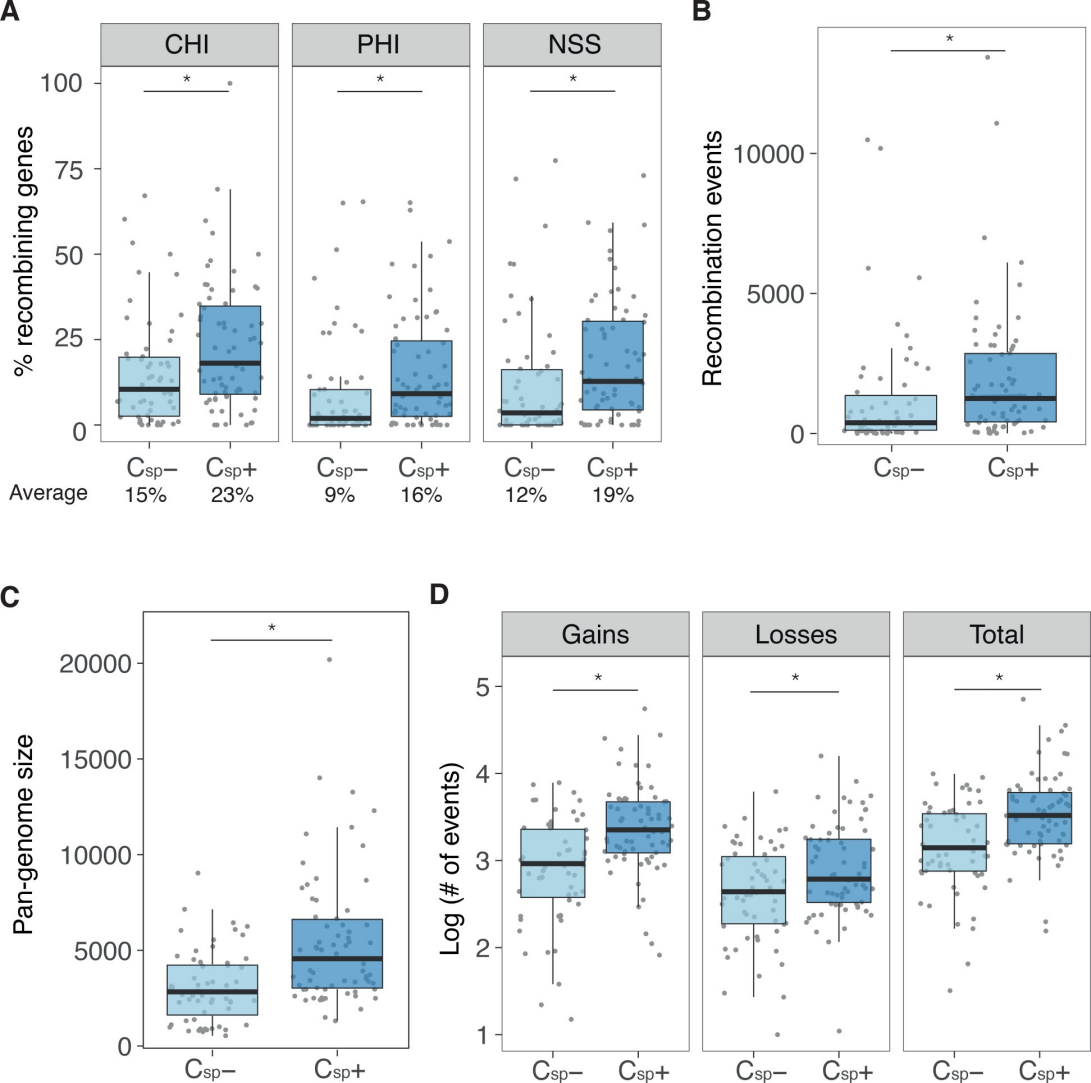
Gene exchange in bacterial species is higher in species coding for capsules. **A**. Percentage of genes for which the null hypothesis of no homologous recombination was refuted by PHI program as measured by excess polymorphism (CHI), by phylogenetic incongruence (PHI) and by neighbour similarity score (NSS). Species with capsules are designated as C_sp_+, *N* = 68; species without capsules are grouped as C_sp_-, *N* = 59. Percentage at the bottom of the panel indicates the average percentage of recombining genes. The median is highlighted by the boxplot. **B**. Number of recombination events as inferred by ClonalFrameML. **C**. Comparison between species with and without capsule in pan-genome size expressed as the number of gene families. **D**. Horizontal gene transfer events as inferred by Count. Events are log_10_-transformed for visual purposes. See S1 Table for the details on the statistical tests. * P < 0.05, GLM. Points represent individual species, and dispersion along the x-axis was done for visualization purposes.

We inferred the core genomes of each species, and found that C_sp_+ have larger core genomes than C_sp_- (S3 Fig). We used the alignments of the families of core genes to quantify homologous recombination (HR) using four methods (PHI, MaxCHI, NSS, ClonalFrameML, see Methods). These methods measure different traits associated with recombination and their joint analysis, if consistent, should provide robust results (see Methods). Indeed, these recombination detection methods produced results that were highly correlated (average Spearman’s ρ = 0.81, all comparisons *P* < 10^−4^, S4 Fig). We show that C_sp_+ species contain a significantly larger proportion of recombining genes (Fig 1A). Additionally, C_sp_+ underwent 1.6 times more recombination events as measured using ClonalFrameML (Fig 1B). We controlled these results with four additional analyses. We first performed the analysis in rarefied datasets, where each species is represented by five random genomes (S5 Fig). We then made the same analyses using species where all genomes either encoded or lacked a capsule locus (N = 110) (S6 Fig). We used generalized linear models to assess if the presence of covariates affected these conclusions (S1 Text, S1 Table). Finally, we controlled the associations for phylogenetic structure (S2 Text, S2 Table). All these analyses confirmed our conclusions, except the latter, where the association was at the borderline of statistical significance (P = 0.078).

We then quantified the diversity of gene families within each species–its pan-genome—and found that C_sp_+ species had 2.1 times larger pan-genomes than C_sp_- (Fig 1C). We used the core genome phylogenetic tree of each species to infer, with birth-death models, the rates of gene gain and loss in the tree. This analysis revealed that C_sp_+ species underwent three times more events of gene gains by HGT (Fig 1D). This was further confirmed using asymmetric Wagner parsimony instead of birth-death models [35] (S5 Fig). As observed for homologous recombination, our results remained significant when controlled for genome size (P = 0. 0104 for pan-genome size and P = 0. 0294 for HGT, GLM) and phylogeny (S2 Text), when using rarefied datasets (S5 Fig), and when using species without polymorphism in the presence of the capsule (S6 Fig).

Because most studies suggesting a negative effect of capsules in genetic exchange focused on naturally transformable species [18–21], we further analysed these results in function of competence for DNA transformation. We selected from our dataset the species known to be naturally transformable according to the literature [36], and compared them with the remaining ones. Bacteria encoding capsules show higher rates of recombination than the others in both groups, but differences between groups are not significant (S2B and S2C Fig).

We conclude that species encoding capsules have larger and more diverse gene repertoires, which change more frequently by horizontal gene transfer, and recombination. These effects are common to multiple methods to define HR and HGT, are robust to the rarefication of the dataset, and to the control by covariates. With the exception of the results for HR, they are also robust to the control by phylogeny.

### Genomes encoding capsule systems have more mobile genetic elements

**Fig 2:**
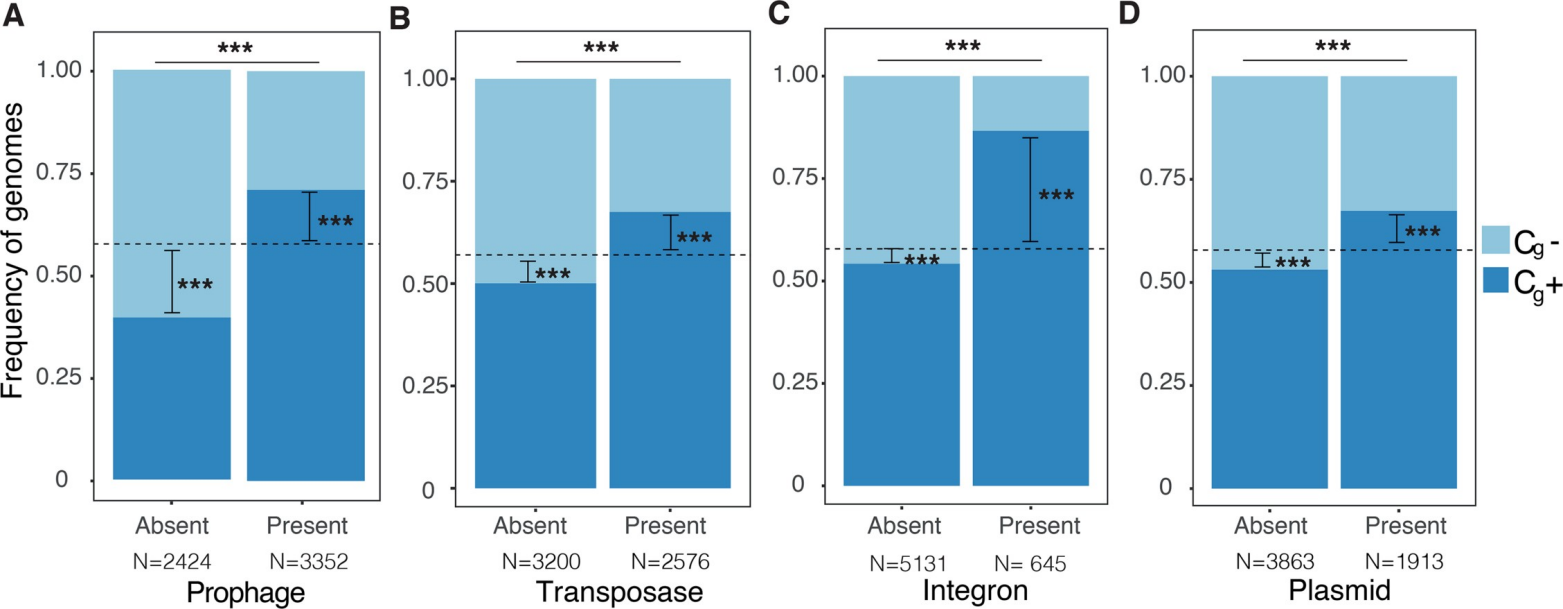
**Co-occurrence between capsule systems and mobile genetic elements; Prophages (A), Transposases (B), Integrons (C) and Plasmids (D).** Stars inside bars represent the result of two-tailed binomial tests to measure the difference between the observed over the expected events, indicated by the dashed line corresponding to the database bias (57%). The stars on top of the bars are the result of dependence tests (χ2 test). All statistics were corrected for genome size and phylogeny (see S3 and S4 Tables for details). *** *P* < 0.001.

If species encoding capsules have higher rates of genetic exchange, by conjugation and transduction, then one would expect them to have more mobile genetic elements (MGEs). To test this hypothesis, we do not need to restrict our analysis to the species with more than four genomes. Instead, we can directly test this at the genome level (indicated by a *g*). We searched over 5000 genomes from more than 2000 species, for loci encoding capsules and for the best known MGEs: prophages, transposases (IS), integrons, and plasmids (see Methods). We classed genomes in those encoding a capsule system (hereafter referred to as C_g_+ by analogy to C_sp_+) and lacking them (C_g_-). The use of all available genomes means that some are much closer than others in our dataset. Since the presence of capsule systems and MGEs across genomes showed some phylogenetic inertia (S3 Table), we controlled the results for this effect using BayesTraits [37]. This was done only for the genomes of Proteobacteria and Firmicutes (73% of the genomes) because deeper phylogenetic trees are hard to estimate accurately. We observed that all MGEs were more likely to be present in genomes that also encode capsule systems (C_g_+) than in the others (C_g_-) (Fig 2), and the control by the phylogeny did not change the conclusions of the analysis (S4 Table).

The analysis above focused on the presence or absence of MGEs in the C_g_+ versus C_g_- genomes. However, C_g_+ also accumulated more MGEs per genome than the other bacteria (S5 Table and S1B Dataset). For the types of elements that are present at an average frequency higher than one in the entire dataset, we computed the association between the number of elements and the presence of a capsule system. In agreement with previous results, these elements are more abundant in C_g_+ (S5 Table). Further, the cumulative size of prophages and plasmids per genome was greater in C_g_+ than in C_g_- genomes (respectively 2.27 and 3.2 times more, S7 Fig and S5 Table). We conclude that C_g_+ genomes are more likely to have MGEs, and in a higher number, than C_g_- genomes.

### Capsule systems are encoded in MGEs

Frequent presence of capsule systems in MGEs could explain the association between the presence of capsule systems and HGT. We started by searching for capsule systems in plasmids, which had previously been described in *Bacillus anthracis* [38–40], and found 225 systems in 163 out of the 4453 plasmids of the database (S6 Table). Thus, one plasmid can code multiple capsule systems. Capsules can be grouped in different types depending on their synthesis pathway; polysaccharidic capsules such as Group I (Wzy-dependent), Group II and III (ABC-dependent), Group IV, and synthase-dependent or proteic poly-γ-d-glutamate capsules (PGA). Their prevalence in plasmids varies markedly: only one Group IV capsule was found on a plasmid (0.15%), whilst 75% of all hyaluronic acid capsules (synthase-dependent) and 20% of all protein capsules were also found within these elements (S8 Fig). Plasmids encoding capsule systems are particularly frequent in Alphaproteobacteria and Firmicutes, but are found in many phyla, including Cyanobacteria or Acidobacteria (S6 Table).

**Fig 3:**
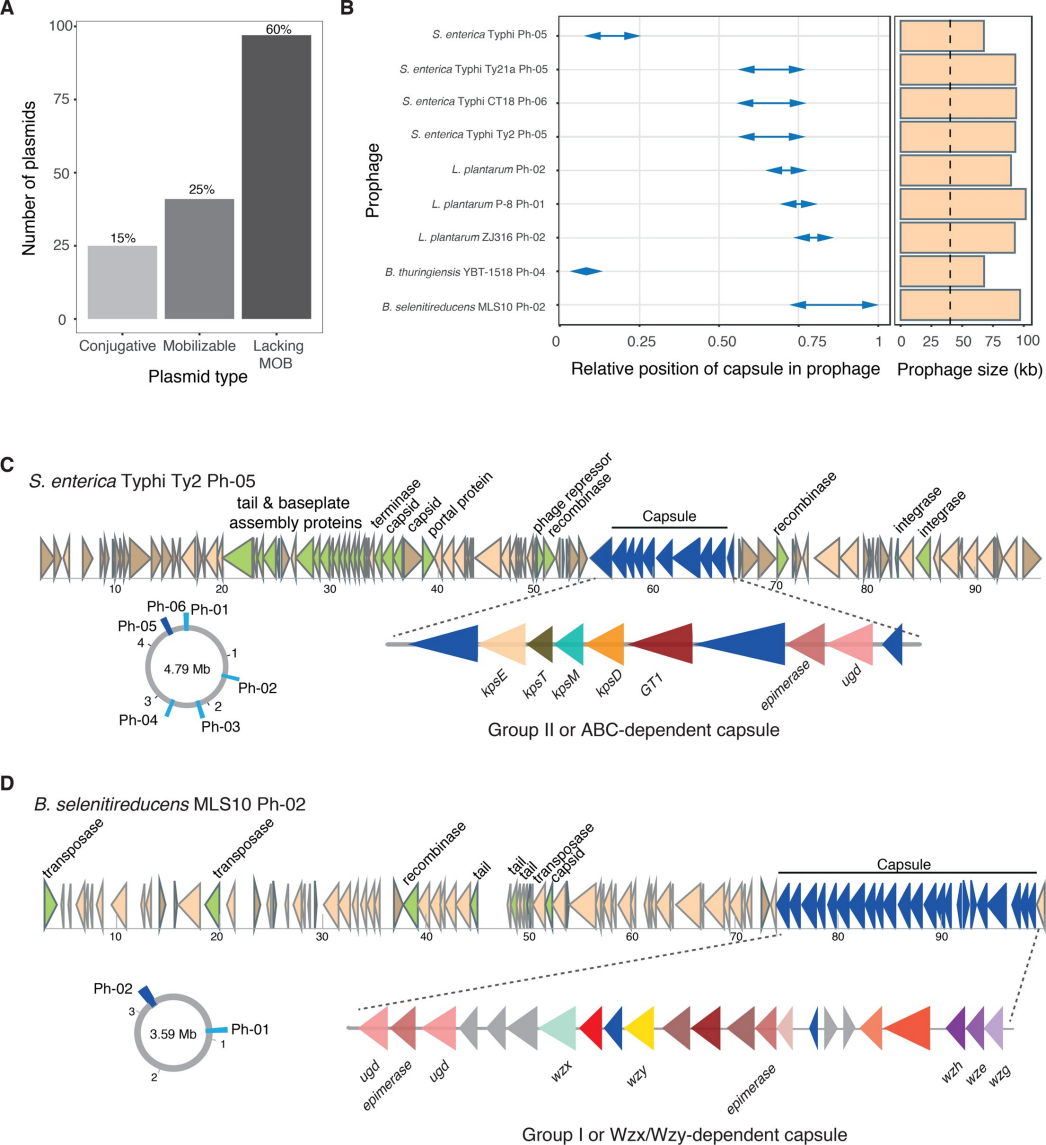
Capsule systems in MGEs. **A.** Number of plasmids encoding capsule systems in function of the type of plasmid (classed in terms of mobility by conjugation). Plasmids lacking MOB may be mobilized by conjugation if they have a compatible *oriT* or mobilizable by other unknown means (*e*.*g*., natural transformation in competent species). **B**. Details of the nine capsules found in prophages. The arrows indicate the relative position and span of the capsule system in each prophage. Right panel indicates the size of each prophage. Dashed line indicated the average size of prophages in the database (40 kb). **C and D.** Details of the prophages and capsule systems from *S*. *enterica* and *B*. *selenitireducens*. Genetic schemes are drawn to scale (kb). In the drawing of the genetic locus of the prophage; genes associated to prophage biology are highlighted in green and capsule genes in dark blue. Circular diagrams represent the genomic localization of all the prophages in both species. The capsule-coding prophage is highlighted in dark blue. In the drawing of the locus of the capsule system, proteins in red-pink tones are associated to sugar modifications and may determine capsular serotype. Gene names are indicated below the arrows. GT1: glycosyl transferase.

We analysed these plasmids in terms of genetic mobility. Those encoding a complete conjugative system were classified as conjugative and those encoding at least a relaxase were classed as mobilizable (as in [41]). The analysis using ConjScan [42] showed that ~40% of the plasmids coding for a capsule were either conjugative or mobilizable (Fig 3A, S6 Table). This distribution is similar to the frequency of these types of plasmids in the database [41]. On the other hand, plasmids encoding capsule systems were larger than expected, given the size of plasmids in the database, showing a median of 224 kb (median of the database: 107kb, P < 0.001, one-sample t-test). This can be explained in part by the size of the capsule locus that can only be encoded in medium sized and large plasmids. Of notice, 40 of the plasmids encoding capsule systems, that is 25%, were larger than 1 Mb and might be regarded as secondary chromosomes. These results show that plasmids often encode capsules, which could explain the high rates of transfer of these loci.

To the best of our knowledge, one single capsule system has been previously identified in a pathogenicity island that could be part of a bacteriophage (henceforth referred to as phage) [43]. All 1943 bacteriophages in our dataset lacked recognizable capsule systems. Yet, unexpectedly, we found a total of 13 capsule systems encoded in regions predicted to be prophages (S7 Table). Manual curation of the dataset of prophages showed that in four cases, capsules were encoded apart from the region between the integrase and the structural genes. In these cases, it is difficult to know if the capsule is part of the phage genome, if it was brought by specialized transduction, or if it is separate from the prophage and the result of an annotation error. As such, these cases were not further analysed. In the remaining cases (*N* = 9), the capsule genes were encoded between the integrase and the structural module, suggesting that the capsule is an integral part of the temperate phage. The four prophages found in *S*. *enterica* are very similar in sequence (S8 Table), and might thus be the result of a single ancestral event of infection. These prophages have a locus encoding a Group II capsule flanked by two recombinases, suggesting that it was a recent accretion to the phage genome. This prophage, also named the large pathogenicity island SPI7, has been experimentally shown to excise, and code for the capsular antigen Vi [43].

The putative capsule-encoding prophages were significantly larger than the average of our dataset (88 kb vs 40kb, one sample t-test, *P* < 0.0001), and were found in the *Salmonella enterica* serovar Typhi (4), and in Firmicutes such as *Lactobacillus plantarum* (3), *Bacillus thuringiensis*, and *B*. *selenitireducens* (Fig 3B, 3C and 3D and S7 and S8 Tables). The capsule types found in prophages represent the most common capsule types, namely Group I and Group II [17]. Taken together, our data shows that capsule systems can spread through a population by different mechanisms of HGT.

### Co-occurrence of capsules with defence mechanisms

In Bacteria, the acquisition of exogenous genetic material is modulated by different defence mechanisms such as restriction–modification systems (hereafter referred to as RMS) that cleave foreign DNA with modification (methylation) patterns that differ from those of the host cell [44] and CRISPR-Cas systems that provide acquired immunity against phages and plasmids [45]. We found no significant co-occurrence between CRISPR-Cas and capsule systems (S9 Fig) nor with the number of spacers (*i*.*e*. length of CRISPR array). This concurs with previous studies that found no association between the frequency of HGT and the presence of CRISPR-Cas systems [46].

**Fig 4:**
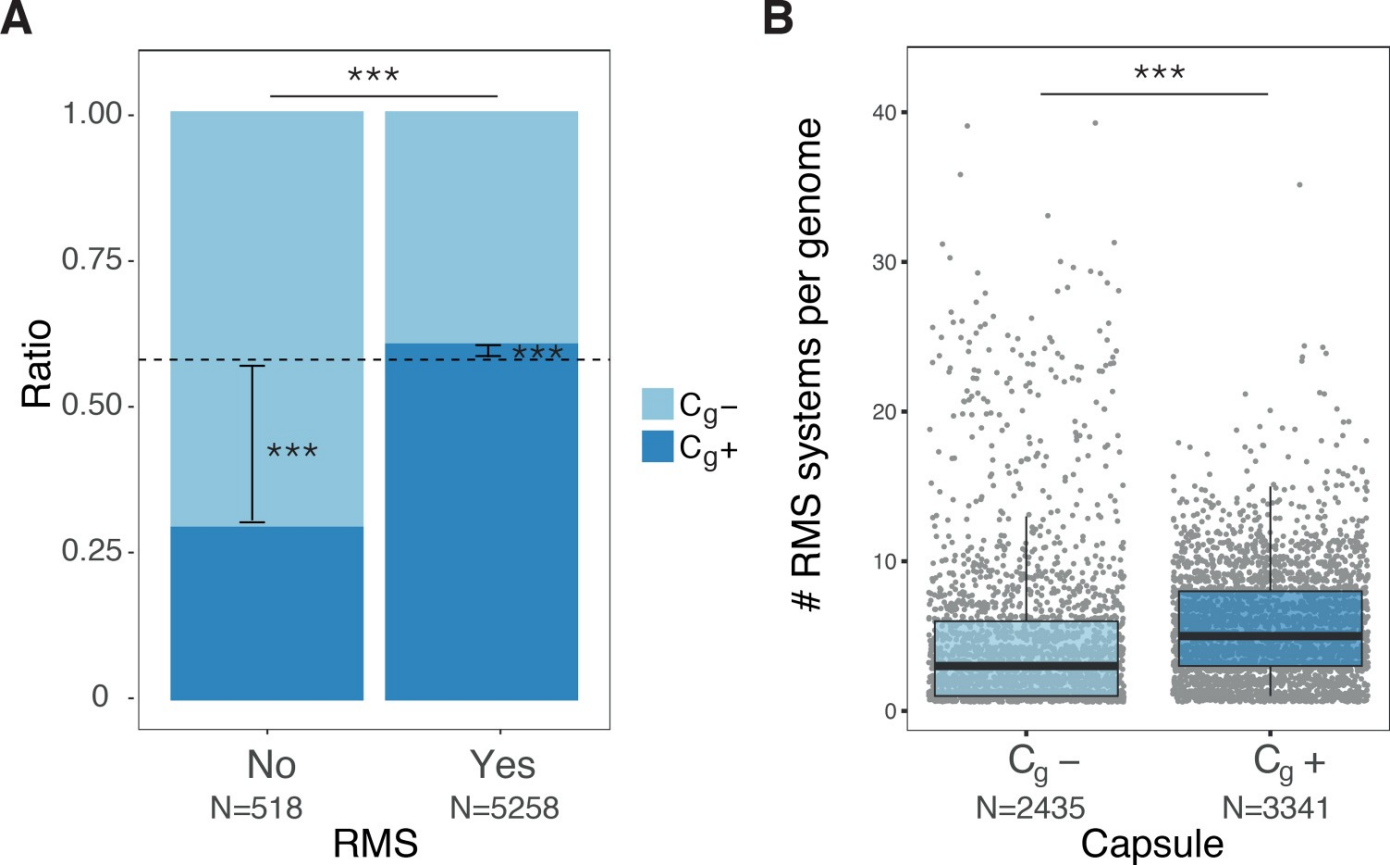
Co-occurrence between capsule systems and RMS systems. **A.** Presence of RMS systems in genomes with and without capsule system (χ^2^ test, and corrected for genome size with *glm*). RMS systems were identified using the highly specific and publicly available HMM profiles in https://gitlab.pasteur.fr/erocha/RMS_scripts. To control for phylogeny we made a complementary analysis restricted to Firmicutes and Proteobacteria. This analysis gave similar results (using BayesTraits, Bayes Factor of 41.3 and 17.2 for Proteobacteria and Firmicutes respectively). Dashed line indicates the ratio of genomes encoding at least one capsule system in the database (57%). **B.** Number of RMS systems in genomes with and without capsule systems. Correction for genome size was performed as above. Phylogeny was taken into account using GEE (P < 0.0001 and P = 0.1 for Proteobacteria and Firmicutes).

It has been previously shown that the distribution of RMS correlates with the presence of MGEs and with higher rates of horizontal gene transfer [47]. This has been interpreted as the result of selection for more RMS in bacteria enduring high rates of infection by MGEs. We thus expect that genomes coding for capsules co-occur more often with RMS. Indeed, our analyses show that the distribution of RMS and capsules systems is strongly correlated (Fig 4A). As previously observed with MGE, there are also significantly more RMS in C_g_+ than in C_g_- (Fig 4B).

### Capsules do not limit the spread of antibiotic resistance

**Fig 5:**
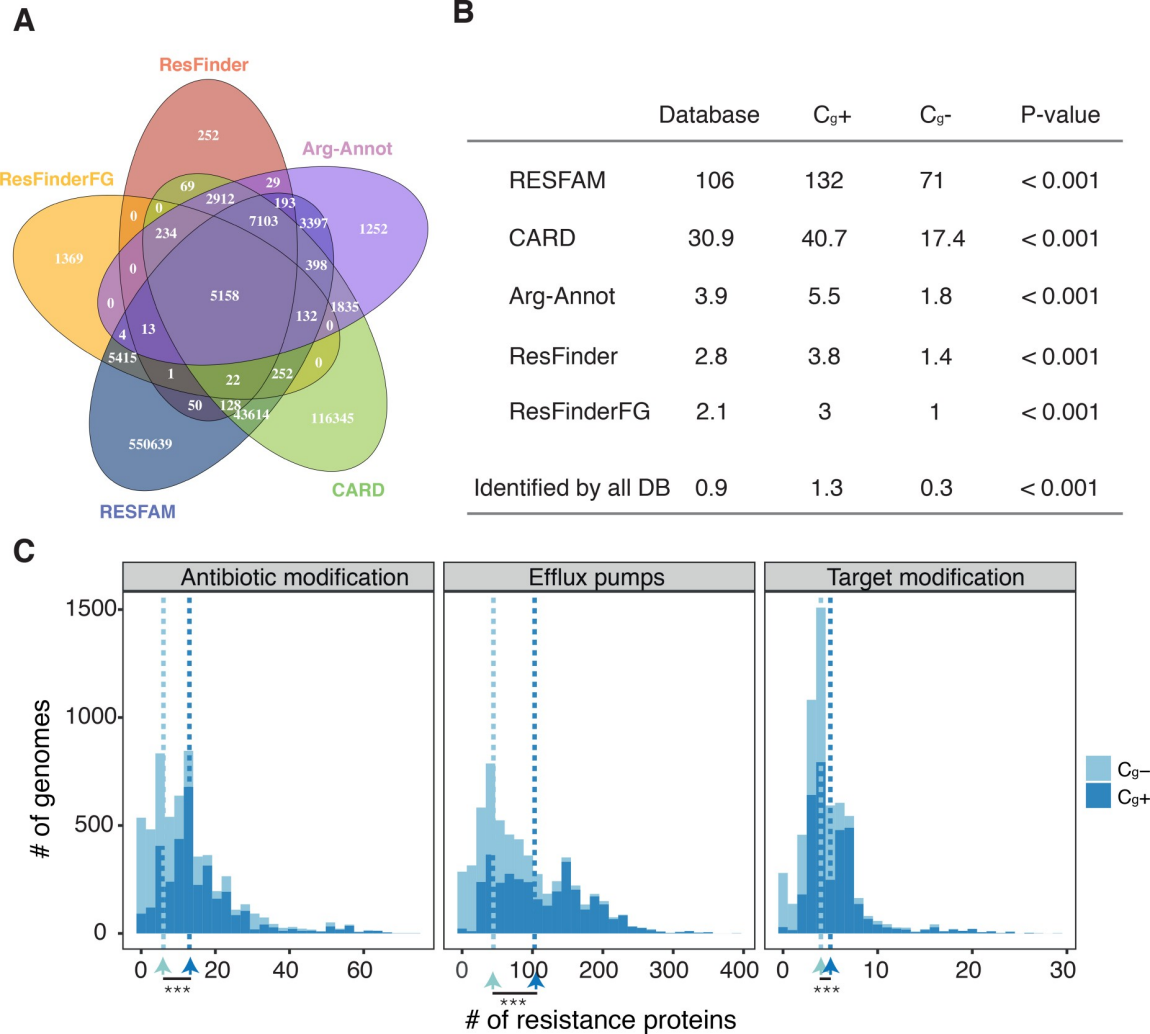
Antibiotic resistance proteins in genomes. Results displayed correspond to those hits with at least 50% of protein identity for all databases (except RESFAM, which is based on HMM profiles). **A**. Venn Diagram showing the total number of genes associated with antibiotic resistance in all C_g_+ according to five different ARG databases. **B**. Mean number of ARGs per genome, for all five databases and the intersect between them (Identified by all DB). *P*-value corresponds to the difference between the mean MGE in C_g_+ and in C_g_- genomes (all corrected for genome size). **C**. Distribution of resistance proteins per genome in function of capsule content classified by resistance mechanisms. These results are based on protein hits from the RESFAM database. Stars indicate significant difference in the median number of resistance proteins, *** P < 0.001.

Our results show that bacterial genomes encoding capsules have more horizontally transferred genes and accumulate more MGEs. It is also well documented that MGEs drive the spread of antibiotic resistance within most lineages of nosocomial pathogens [48, 49]. Furthermore, by favouring HGT, capsules could enhance the acquisition and spread of antibiotic resistance genes. We thus hypothesized that bacteria encoding capsules could also encode more antibiotic resistance genes (ARGs). We searched for capsule systems in the six species of notorious ESKAPE pathogens, the leading cause of nosocomial infections throughout the world [50]. All of them encoded capsule systems in more than 80% of genomes. We also identified capsule systems in most genomes of 10 out of the 12 clades included in the WHO list of bacterial clades in urgent need of novel antibiotics (all except *Neisseria gonorrhoeae* and *Helicobacter pylori*). Then, to detail the association between capsule systems and ARGs, we searched all genomes in our dataset for the latter using the RESFAM database [51]. We identified 91% more genes associated with ARG profiles in C_g_^+^ than in C_g_- (*P* < 0.000, controlled for genome size). Since ARGs are difficult to identify, we confirmed this trend by further analysing our dataset with four other reference databases (CARD, Arg-Annot, ResFinder and ResFinderFG, [52–54]), with the intersection of all of them (Fig 5A and 5B), and by varying the protein sequence similarity cut-off (50% or 80%, Fig 5B). All these analyses showed a significant over-representation of ARGs in C_g_+, even if the number of identified genes differed markedly across them.

Antibiotic resistance is commonly classed according to three major mechanisms: active efflux of the antibiotic to the outside of the cell, enzymatic modification of the antibiotic, and mutation of the antibiotic target (Fig 5C). We focused on the RESFAM database and analysed separately the ARGs associated with each of these mechanisms. They were all more abundant in C_g_+ than in C_g_- (Fig 5C). This difference was particularly large for efflux pumps, which were over-represented in C_g_+ at a larger extent than the others (two-tailed binomial test P < 0.001). Hence, the presence of capsule systems is associated with that of antibiotic resistance genes, and especially those involving efflux pumps.

## Discussion

Capsules play important roles in inter-species competition, survival under harsh conditions, and niche colonization [15, 17]. Bacterial adaptation under such conditions is accelerated by the exchange of genetic information between cells [55, 56]. Several previous works have shown that the latter drives the rapid evolution of capsules by horizontal gene transfer and recombination [4, 5, 33, 57]. This results in a conundrum. On one hand, both genetic exchanges and capsules could be adaptive under similar circumstances (and capsule systems themselves are often exchanged between cells). On the other hand, it has been proposed that capsules decrease the rates of genetic exchange [21, 23, 26], presumably implicating a decrease in the rates of bacterial adaptation and of capsule diversification. Here, we show that this implication is not valid using multiple lines of evidence, where the presence of a capsule locus is positively associated with the frequency of genetic exchanges either by recombination or horizontal gene transfer, with larger pan-genomes, more integrons, more plasmids, more prophages, and more ISs. Some of these MGEs encode capsule systems. These bacteria also tend to show higher rates of HR in the core genome, independently of being naturally transformable or not. The consistency of all these analyses shows that the effect we measure is general and not limited to a set of mechanisms or MGEs. Hence, bacteria encoding capsule systems tend to display higher rates of genetic diversification than the others, even if certain bacteria lacking capsules can diversify rapidly (e.g., *Neisseria gonorrhoeae* and *Helicobacter pylori*).

These results are in agreement with the hypothesis that capsules and genetic exchanges are adaptive under similar circumstances, and that the latter are important for the genetic diversification of capsular loci. However, they also raise the question of what mechanisms drive the positive association between genetic exchanges and the presence of the capsule. We propose four alternative scenarios: (i) transfer takes place when bacteria are not expressing the capsule, (ii) the presence of capsules and the rates of genetic exchange co-vary indirectly by way of their interaction with other mechanisms, (iii) increased genetic exchanges directly increase the frequency of capsule loci, or (iv) the presence of capsules directly increases genetic exchanges.

First, transfer between bacteria could take place when capsules are not expressed. A model mimicking biofilm formation during pneumococcal carriage reported higher efficiencies of natural transformation and lower levels of capsule expression in this species [22]. Thus, cells could alternate between periods of capsule expression and low transfer and periods where they lack a capsule and favour genetic transfer. Alternatively, some cells in the population may lack a capsule, either because it is subject to phase variation [58, 59], gene loss [60, 61], or to stochastic phenotypic heterogeneity at the cellular level [62], and these cells may account for a large fraction of genetic exchanges. Such switching phenotypes emerge easily as a response to fluctuating environments and allow faster adaptation whilst minimizing capsule cost [63]. A problem with these explanations is that capsulated bacteria have more genetic exchanges than non-capsulated bacteria. If these exchanges take place between a small fraction of the population, or in short periods of time, then exchange rates in bacteria encoding but not expressing capsules must be exceedingly high compared to those of bacteria lacking capsular loci. It seems more parsimonious to consider the possibility of direct or indirect associations between capsules and genetic transfer.

Second, the association of genetic exchange with the presence of capsule loci could be explained indirectly by way of their positive effect on the rates of adaptation [64, 65]. Bacteria with broad environmental ranges are expected to face higher rates of genetic exchanges and most have been shown to encode capsules [17]. The two traits are expected to show similar responses to environmental cues. For example, antibiotics, such as beta-lactams, induce the transfer of prophages and conjugative elements and the expression of integrons [66–68], thus increasing the rates of genetic exchange in conditions that have been shown to raise the expression of capsules [69]. Furthermore, capsulated bacteria have higher survival rates relative to the other bacteria in the presence of antibiotics [9]. The combination of increased survival and presence of MGEs in bacteria encoding capsules might increase the rates of HGT in capsulated cells under antibiotics (and other equivalent stressors). In *S*. *pneumoniae*, where several laboratory and epidemiological studies suggested a negative association between natural transformation and capsule production [19, 21, 23], there is a positive correlation between capsule size and genetic exchange during carriage, because large capsules are associated with longer carriage and thus increase the chances of genetic exchanges [24].

Third, genetic exchanges are needed for the acquisition and diversification of capsule operons [4, 33, 57], and bacteria engaging in more exchanges are thus more likely to encode a capsule. Capsule diversification involves recombination, gene insertion, loss, and inactivation, often mediated by transposable elements [5, 70, 71]. A constant input of novel genes to the loci may be required to maintain its function. As a consequence, bacteria with very low rates of transfer might be less likely to encode a capsule because of the lower rate of (re-) acquisition of the locus (or parts of the locus).

Fourth, capsules might directly favour genetic exchanges [24, 72]. Most data on *S*. *pneumoniae* suggests the opposite [19, 21, 23], although in *Haemophilus influenzae* transformation and plasmid conjugation seem to be less affected [31, 73], and in *Pseudomonas aeruginosa*, conjugation seems unaffected by the presence of a capsule [74]. Further, the role of capsules in phage infection seems to be strain-dependent [25–27]. One could however speculate that capsules by producing a structured environment would favour conjugation (usually less efficient in well-mixed environments) and transduction (by producing patches of closely related lysogens) in natural complex communities.

A caveat of this study, in assessing the possibility of a direct positive effect of capsules on the rates of genetic exchanges is that we dispose of little experimental evidence on whether most of these species are able to express and produce a capsule in the environments in which HGT is highest. We also ignore how the capsule is regulated (genetically or epigenetically) in such environments. Therefore, more experimental work beyond the *S*. *pneumoniae* model is needed.

Our study shows that the presence of capsule systems is associated with rapid genome diversification driven by genetic exchanges with other bacteria. Although under extremely stressful conditions leading to reduced metabolic rate (*i*.*e*. dormancy), genetic exchanges might be hampered independent of the presence of capsules, the latter most likely increase resilience and persistence in the environment. Thus, bacteria with capsules enjoy a triple advantage: they are more protected from environmental challenges, capsule-mediated survival expands the time span available for the acquisition of adaptive traits, and the probability of acquisition of the latter is higher because of the frequent genetic exchanges between these bacteria. Even if the costs of capsule production can be very high [28, 63], these advantages may contribute to explain why genomes encoding capsule systems encode more ARGs and are the majority of the most worrisome facultative and nosocomial pathogens.

## Materials and methods

### Data

The genome database was composed of 6219 chromosomes and 4453 plasmids of 5576 bacterial and 213 archaeal fully sequenced genomes representing 2437 species downloaded in November 2016 from NCBI RefSeq (ftp://ftp.ncbi.nih.gov/genomes/). The sequences and corresponding annotations of 1943 complete bacteriophage genomes were retrieved from GenBank in September 2016.

### Identification of capsules

We used CapsuleFinder as published in [17] to search for Group I (or Wzy-dependent), Group II and III (ABC-dependent), Group IV (subtypes e, f and s), synthase-dependent (subtypes cps3-like and hyaluronic acid) and PGA (Poly-γ-d-glutamate) capsules in the genome database. This allowed the detection of 5596 systems in 3341 genomes (57% of the database) belonging to 1273 different species (S10 Fig). We also ran Group IV capsule models without the gene *wzx* considered forbidden (*ie* incompatible with Group IV capsule). This did not have any impact in our results as it did not alter whether a species was classified as C_sp_+ or C_sp_-.

The identification of capsules was performed at the genome level (C_g_) whereas the inference of the core and pan-genome, and thus of HGT and HR, were performed at the species level (C_sp_), when at least five complete genomes were available. Such analyses required a classification of species into those encoding capsules (C_sp_+) and those lacking them (C_sp-_). In the vast majority of cases, the different strains of a species had the same capsule phenotype (that is, the frequency of genomes with at least one capsule) (S10B Fig). When they didn't, to account for the frequency of the rare variant: if more than 80% of the species concurred (in presence or absence of the capsule) they were classed according to the predominant trait (S10B Fig). Otherwise, we excluded the species from further analysis. This led to the exclusion of 10 out of 137 species leading to the use of 10% of species in the core/pan-genome related analyses. All analyses were repeated using only species for which 100% of the genomes concurred in the presence or absence of capsule. This resulted in a further reduction of the dataset from 127 to 110 species. Nevertheless, this did not alter the trends observed between capsule and genetic transfer (S6 Fig).

### Identification of MGEs

*(i) Prophages* were detected using Phage Finder v4.6 (using default parameters, including “plasmid” replicons). We removed overlapping prophages selecting the longest prophage (only 26 cases), which resulted in 9,876 elements. Elements larger than 18kb were considered as prophages (8,385 elements), the smaller elements as putative remnants prophages. The 13 prophages with detected capsule systems were manually curated to ensure that they were *bona fide* prophages. This resulted in the exclusion of four putative prophages. *(ii) Integrons* were detected using IntegronFinder as described in [75]. *(iii) Transposases* were identified using HMM profiles as described in [76]. *(iv) Plasmids* were retrieved from the GenBank files and the annotations were used to distinguish them from secondary chromosomes. To detect whether plasmids were conjugative, mobilizable, or none of the two, we used CONJscan [42]. We used default settings, except that we set inter_max_gene_space to a very high value (1500) between the relaxase, VirB4 and the coupling protein because it is more appropriate for very large plasmids. Mobilizable plasmids were those in which the relaxase and the coupling protein co-localized but VirB4 was absent.

### Detection of antibiotic resistance genes

To analyse the presence of genes involved in antibiotic resistance in the genome database, we used the full RESFAMv1.2, CARD, Arg-annot, Resfinder v3.0 and ResfinderFGv1.0 databases [52–54]. The RESFAM database was queried with the–*cut_ga* option (curated for accuracy). The results were filtered to select those having E-values lower than 10^−20^ for the full sequence and 70% coverage of the profile. The other databases were searched for hits with a minimum e-value of 10^−20^ and at least 70% coverage of the profile. All results displayed are based on the RESFAM database unless stated otherwise. We performed all tests in triplicate without using a cut-off for protein identity and with 50% or 80% cut-off. This did not alter the results qualitatively.

### Identification of core genomes and pan-genomes

We identified a preliminary list of orthologs between pairs of genomes as the list reciprocal best hits using end-gap free global alignment, between the proteome of a pivot and each of the other strains proteome (as in [76]).

Hits with less than 80% similarity in amino acid sequences or more than 20% difference in protein length were discarded

The list of orthologs was then refined for every pairwise comparison using information on the conservation of the genetic neighbourhood. Thus, positional orthologs were defined as bidirectional best hits adjacent to at least four other pairs of bidirectional best hits within a neighbourhood of 10 genes (5 upstream and 5 downstream). These parameters (four genes being less than one-half of the diameter of the neighbourhood) allow retrieving orthologs on the edge of rearrangement break-points and therefore render the analysis robust to the presence of rearrangements. Finally, the core genome of each species was defined as the intersection of pairwise lists of positional orthologs. The core genome only included single-copy genes. The inclusion of paralogs could lead to confound effects of recombination with foreign DNA with intra-chromosomal recombination.

We imposed an 80% similarity threshold to avoid mixing paralogs or xenologs. To verify that this threshold is not too stringent–that it refuses few true orthologs—we computed the distribution of sequence similarity between pairs of orthologs of the core genome of each species. These distributions showed that values were in general very high, with the average of the species average similarity ranging between 97.4% and 99.99% (mean 99.3). The median values are very similar to the averages, the minimal value being 98.2% (overall median: 99.5). To check that the tail of the distribution was not leading to the spurious exclusion of many fast-evolving proteins, we computed the percentiles 1% and 5% of the values of sequence similarity for the pairs of orthologs for each species. On average, the 1% percentile was at 93% sequence similarity, whereas the 5% percentile was at 97% similarity (meaning that on average 95% of the orthologs are more than 97% similar in protein sequence). Both values are very far from the threshold of 80% similarity. Actually, only one species had the 5% percentile at less than 90% similarity (S11 Fig). This strongly suggest that the threshold of 80% sequence similarity does not lead to the exclusion of a significant number of orthologs.

Pan-genomes are the full complement of genes in the species and were built by clustering homologous proteins into families for each of the 127 species. We determined the lists of putative homologs between pairs of genomes (including plasmids) with MMseqs2.0 [77], by keeping only hits with at least 80% identity and alignment covering at least 80% of both proteins. Proteins were clustered by single-linkage.

### Phylogeny of core genomes

We built core genome trees for each species using a concatenate of the multiple alignments of the core genes (aligned with MAFFT v7.305b ([78] using default settings). Each species’s tree was computed with IQ-Tree v1.4.2 [79] under the GTR model and a gamma correction (GAMMA) for variable evolutionary rates. We performed 1000 ultrafast bootstrap experiments (options–*bb* 1000 and–*wbtl*) on the concatenated alignments to assess the robustness of the topology of each species’s tree. The vast majority of nodes were supported with bootstrap values higher than 90%. We inferred the root of each phylogenetic species’s tree using the midpoint-rooting approach of the R package “phangorn” v1.99.14 [80].

### Inference of homologous recombination (HR)

We inferred events of homologous recombination on the multiple alignments of the core genes of each species using ClonalFrameML (CFML) v10.7.5 [81] with a predefined tree (*i*.*e*. the species’s core genome tree), default priors R/θ = 10^−1^, 1/δ = 10^−3^, and ν = 10^−1^, and 100 pseudo-bootstrap replicates, as suggested by the authors. Mean patristic branch lengths were computed with the R package “ape” v3.3, and transition/transversion ratios were taken from the results of IQ-TREE mentioned above to infer the core genome trees. The priors estimated by this mode were used as initialization values to rerun CFML under the “per-branch model” mode with a branch dispersion parameter of 0.1. ClonalFrame and ClonalFrameML were built to analyze recombination from outside of the clade under analysis [82]. Hence, they may lack power to detect recombination within species. This problem is explicitly tackled by the authors of ClonalFrame [82] that show that it identifies recombination events very accurately when used at the species-level (90% accuracy), even if it may miss a significant number of events. This has led to the frequent use of this software for species-level analysis in a way similar to the one done here (e.g., [83–85]).

We also inferred the presence of recombination in the alignments of core genes with the maximum χ^2^ (MaxCHI), the neighbour similarity score (NSS) and with the pairwise homoplasy index (PHI) with 10,000 permutations using PhiPack [86]. For all three cases, we used as evidence of recombination the threshold given by P<0.05. These programs measure in different ways the existence of recombination in a multiple alignment. They do not infer individual events of recombination nor recombination rates (like CFML).

All analyses of recombination were made on the core genomes of the full datasets and on the core genomes of the rarefied datasets.

### Inference of horizontal gene transfer

We assessed the dynamics of gene family repertoires using Count [87] and as described in [47]. Briefly, this program models the gains and losses of gene families, while accommodating rate variations across phylogenetic lineages and across families. The analysis starts with the estimation of the parameters of the model by maximum likelihood using the pan-genome matrix of gene presence and absence (0/1). Count then uses these parameters to calculate the expected size of each family in every internal node of the species tree. It also computes the expected number of gain, loss, expansion, and contraction events along each branch. Rates were computed with default parameters, assuming the Poisson family size distribution at the tree root, and uniform gain, loss, and duplication rates. One hundred rounds of rate optimization were computed with a convergence threshold of 10^−3^. After optimization of the branch-specific parameters of the model, we performed ancestral reconstructions by computing the branch-specific posterior probabilities of evolutionary events, and inferred the gains in the terminal branches of the tree. The analysis was performed on a matrix of presence-absence of gene families. Hence, duplications were not taken into account.

### 16S Phylogeny

16S rRNA of the 5776 genomes was detected using the RNammer 1.2 software [88] with the options–S set to *bac* and the–m to *ssu*. We then selected the first entry per genome and aligned them using the secondary structure models with the program SSU_Align v0.1.1 (http://eddylab.org/software/ssu-align/). Badly aligned positions were eliminated with *ssu-mask*. The alignment was trimmed with trimAl v1.2rev59 [89] using the option -noallgaps to delete only the gap positions but not the regions that are poorly conserved. The 16S rRNA phylogenetic tree was inferred using IQTREE v.1.5.3 [79] under the GTR+I+G4 model with the options–*wbtl* (to conserve all optimal trees and their branch lengths), and–*bb 1000* to run the ultrafast bootstrap option with 1000 replicates.

### Firmicutes and proteobacteria trees

Trees were built as described in [90]. Briefly, we built the sets of families of orthologous genes that were present in more than 90% of the genomes of Firmicutes (N = 1189) and Proteobacteria (N = 2897) larger than 1 Mb available in the GenBank RefSeq dataset indicated above. Lists of orthologs were identified as reciprocal best hits using end- gap free global alignment, between the proteome of a pivot and each of the other strain’s proteomes. *Escherichia coli* K12 MG1655 and *Bacillus subtilis* str.168 were used as pivot for each clade. Hits with less than 37% similarity in amino acid sequence and more than 20% difference in protein length were discarded. The persistent genome of each clade was defined as the intersection of pairwise lists of orthologs that were present in at least 90% of the genomes representing 411 families for Firmicutes and 341 for Proteobacteria.

We inferred phylogenetic trees for each clade from the concatenate of the multiple alignments of the persistent genes obtained with MAFFT v.7.205 (with default options) and BMGE v1.12 (with default options). Missing genes were replaced by stretches of "-" in each multiple alignment. This approach results in a small number of genomes that lack many of the orthologs and thus have many gaps in the concatenate alignment. These bacteria typically have very small genomes and correspond to endosymbionts. We removed 1% of the genomes with most gaps (12 Firmicute and 30 Proteobacteria) because these might lead to poor phylogenetic inference. As a result, we obtained concatenate alignments that had a maximum of 18% (Firmicutes) and 23% (Proteobacteria) of gaps in a given genome. These were extreme values. On average, we had 3.35% and 2.76% gaps for Proteobacteria and Firmicutes, respectively. Adding a few "-" has little impact on phylogeny reconstruction [91]. The trees of the phyla were computed with FastTree v2.1 under LG model [92]. In both cases, the LG model had lower AIC than the alternative WAG model. We made 100 bootstraps by using phylip’s SEQBOOT to generate resampled alignments and the n intree1 options of FastTree.

### Statistics

All basic statistics were performed using R v 3.3.2. (*i) Statistics between two variables*. Statistics between two variables, except those to control for phylogeny, were done using standard non-parametric tests. *(ii) Controls for covariates*. We controlled the rates of HGT, HR and pan-genome size with relevant variables (S1 Table). This was done using generalized linear models (distribution Binomial, link function logit) where the presence/absence of the capsule was the dependent variable and the focal and control variables were independent variables. We fitted the model and assessed the relevance of the focal independent variable by testing if the parameter estimate for the variable was significantly different from zero (when the overall model had an R^2^ significantly higher than zero, which was always the case). *(iii) estimate of Pagel's Lambda*. The presence of phylogenetic signal in the evolution of traits was estimated with Pagel’s lambda using the *phylosig* function of the *phytools* package v.0.5–20 for R [93] and the aforementioned 16S rRNA phylogenetic tree. *(iv) Controls for phylogenetic dependence between binary and continuous variables*. The associations between the capsule and the focal variables obtained in the analyses of pan- and core genomes (HGT, HR and pan-genome size) were controlled for phylogeny using the 16S rRNA phylogenetic tree using Phylogenetic Generalized Linear Mixed Models [94], where the presence of the capsule was the dependent variable and the focal variable the independent one (as for the controls for co-variates). For this, we used the function binaryPGLMM with default parameters from ape v5.2 [95]. *(v) Controls for phylogenetic dependence among binary variables*. Co-occurrence of capsule and MGE and bacterial defence systems were only studied in Bacteria due to the little data available on Archaea. We used BayesTraits v.2.0 [37] to test the correlations among capsule systems and presence of MGEs and RMS. For this, we used the core genome trees of the Firmicutes and the Proteobacteria (see above). The genomes in these two phyla represent 73% of our database (*N* = 4084). We ran two models (Independent and Dependent) in MCMC mode (priorAll exp 10) and computed the Bayes Factor (BF = 2(harmonic mean (dependent model)—harmonic mean (independent model)). These tests were performed with 100 bootstrap trees and the median Bayes Factor was computed. To test the correlations among capsule systems and the amount of MGEs and RMS we ran the function *compar*.*gee*, a generalized estimating equation from the R package ape, on 100 bootstrap trees. The distribution of P-values was plotted and the median calculated.

## Supporting information

S1 Text Controls for the analyses of recombination. (DOCX)

S2 Text Controls for phylogenetic inertia. (DOCX)

S1 Fig Cladogram of analysed bacterial species. The tree was built using the 16S rRNA sequences of 122 bacterial species. From the inside to the outside: squares indicate the presence (full) or absence (empty) of capsule systems in all genomes of each species; rectangles represent the core (orange) and pan-genome (red) size, the number of homologous recombination events (blue) and total number of HGT events (green) (calculated as the sum of all gains and losses). Legends show minimum, median and maximum values. The circles along the branches are color-coded and proportional to bootstrap values.
(DOCX)

S2 Fig Species recombination in function of capsule and competence for natural transformation. **A.** Co-occurrence between the presence of capsule and competence system in our species database. Dashed line indicates the ratio of species encoding at least one capsule system in the database (51%). Pearson's χ^2^ test with Yates' continuity correction. *N*.*S*. = not significant. **B**. Number of recombination events as inferred by ClonalFrameML. **C**. Percentage of genes for which the null hypothesis of no homologous recombination was refuted by PHI program as measured by excess polymorphism (CHI), by phylogenetic incongruence (PHI) and neighbour similarity score (NSS).
(DOCX)

S3 Fig Core genome size of species with (C_sp_+) and without capsule(C_sp_-). Core genome size is expressed as the number of gene families present in all genomes of a given species (*N* = 127, ** P < 0.01, logistic regression controlled by genome size, S1 Table).
(DOCX)

S4 Fig Heatmap representing the correlations between the different measures of recombination used in this study. All correlations are statistically significant, P < 0.001.
(DOCX)

S5 Fig Gene exchange in bacterial species is higher in species coding for capsules as calculated with the rarefied sets (five randomly chosen genomes per species). **A**. Percentage of genes for which the null hypothesis of no homologous recombination was rejected by the PhiPack program as measured by the tests: CHI, PHI, and NSS * P < 0.05, GLM. **B**. Number of recombination events as inferred by ClonalFrameML, ** P < 0.01, GLM. **C**. Comparisons of pan-genome size (expressed as the number of gene families) between species with and without capsule. **D**. Horizontal gene transfer as estimated by Wagner parsimony method (MRCA; most recent common ancestor). **E.** Horizontal gene transfer events as inferred by Count using birth-death models. * P < 0.05, GLM. Points represent individual species, and dispersion along the x-axis was done for visualization purposes.
(DOCX)

S6 Fig Gene exchange in bacterial species calculated using only species with all genomes belonging to C_sp_- or C_sp_+. This resulted in a reduced dataset with 110 species, of which 59 were C_sp_- and 51 C_sp_+. **A**. Percentage of genes for which the null hypothesis of no homologous recombination was rejected by the PhiPack program as measured by the tests: CHI, PHI, and NSS * P < 0.05, GLM. **B**. Number of recombination events as inferred by ClonalFrameML. **C**. Comparisons of pan-genome size (expressed as the number of gene families) between species with and without capsule. **D**. Horizontal gene transfer events as inferred by Count using birth-death models. * P < 0.05, GLM. Points represent individual species, and dispersion along the x-axis was done for visualization purposes.
(DOCX)

S7 Fig Increased amount of foreign DNA in genomes coding for capsules. Cumulative size of all prophages (**A**) and plasmids (**B**) per genome, log_10_-scale. Statistical test corresponds to a logistic regression controlled by genome size *** P <0.001
(DOCX)

S8 Fig Capsules encoded in plasmids. Distribution of capsules in the chromosome and plasmids. Dashed line indicates the average across the whole dataset (~4%).
(DOCX)

S9 Fig Co-occurrence between capsule systems and CRISPR-Cas systems. CRISPR-Cas were identified as described in [96]. *N*.*S*. = not significant, Pearson’s χ^2^ test.
(DOCX)

S10 Fig Summary statistics of capsule systems detected in the database. **A.** Number of systems of each capsule type detected in the dataset of 5576 genomes. Numbers on top of bars indicate percentage of each capsule type. **B.** Distribution of genomes encoding a capsule (C_g_+) in each bacterial species for which we dispose of more than 4 genomes. We discarded species with more than 0.2 and less than 0.8 C_g_+.
(DOCX)

S11 Fig Percentage of sequence similarity for the 5% percentile of the core genome. (DOCX)

S1 Table Statistic details for rates of genetic exchange and genetic richness. We performed a logistic regression to control for genome size and other associated variables to the response trait.
(DOCX)

S2 Table Phylogenetic inertia of gene transfer exchanges and capsule systems. We estimated the phylogenetic inertia of several genetic transfer measures using Pagel’s **λ** included in the *phytools* package and a 16SrRNA phylogenetic tree. The null hypothesis is **λ** = 0 (no phylogenetic effect).
(DOCX)

S3 Table Phylogenetic analysis of the distribution of MGEs and capsule systems. We estimated the phylogenetic inertia of the presence of capsules and MGE in genomes using Pagel’s **λ** included in the *phytools* package and a 16SrRNA phylogenetic tree. The null hypothesis (**λ** = 0, no inertia) was always rejected.
(DOCX)

S4 Table Phylogenetic corrections for association between MGEs and capsule systems. To test for the co-occurrence of MGE and capsule in a genome, we used BayesTraitsv3 (see methods) to calculate the Bayes factor. Bayes Factors can be interpreted as follows: <2 weak evidence, >2 positive evidence, 5–10 strong evidence, and >10 very strong evidence. The lower evidence for Firmicutes may be associated with the smaller sample size. Genomes of Firmicutes (N = 1189) and Proteobacteria (N = 2897).
(DOCX)

S5 Table Average number of mobile genetic elements per genome. Only genomes with at least one MGE were taken into account. *P*-values corresponds to the test of difference between the mean number of MGEs in Cg+ and in Cg- (all corrected for genome size and phylogeny, see S3 Table for details).
(DOCX)

S6 Table List of plasmids with capsule systems. (DOCX)

S7 Table List of capsule systems found in prophages. (DOCX)

S8 TableOrthologous proteins and identity between the *Salmonella enterica* prophages.**A.** Number of orthologous proteins between the prophages in *Salmonella* bearing a capsule operon. Threshold for orthologous genes was set to 80% similarity. B. Identity (lower triangle) was calculated for the 59 proteins common to all prophages for all pairwise comparison using *needle* (Needleman-Wunsch) from the EMBOSS package v6.6.0.0 with default options (-gapopen 10.0 -gapextend 0.5) using the proteic sequence. Weighted gene repertoire relatedness (wGRR, upper triangle) was calculated as $\sum_{i=1}^{M}\frac{S_{\left(Ai,Bi\right)}}{min(\eta_{A},\eta_{B})}$, with S_(Ai,Bi)_ representing the similarity score of the pair i of homologous proteins shared by phage A and phage B (bi-directional best hit), M the total number of homologs between phages A and B and *η_A_* and *η_B_* the total number of proteins of phage A and B, respectively.(DOCX)

S1 Dataset Raw data used in this study. **A.** Species data. This file corresponds to the data represented in Fig 1 and describes the average genome size, core and pan-genome sizes, recombination events and horizontal gene transfer events for each of the 127 species. The number of genomes, the percentage of capsulated genomes as well as their clade is indicated. **B.** Genome data. This file corresponds to the data represented in Fig 3 and details the accession numbers, species classification, capsule status and number of mobile genetic elements for each of the 5776 genomes used.
(XLSX)
