# Supplementary material for: Genetic exchanges are more frequent in bacteria encoding capsules
Source: PLoS Genet. 2018 Dec 21;14(12):e1007862. doi: 10.1371/journal.pgen.1007862 (PMC6322790; doi:10.1371/journal.pgen.1007862)
Supplement: S1 Text — (DOCX) [file pgen.1007862.s001.docx]

**Text S1. Controls for the analyses of recombination.**

The association between homologous recombination events and presence of a capsule system, may be affected by several factors, such as the phylogenetic depth of the species, the number of genomes, and the number of core genes used to detect recombination. As a proxy of the first, we used the average tip to root distance of the species trees. This value was not significantly different between C_sp_+ and C_sp_- (P=0.15, Wilcoxon test). We showed that the number of genomes used per species was almost identical between C_sp_+ and C_sp_- (not significantly different, see Main text). In contrast, the number of core genes was higher in C_sp_+ than in C_sp_- (P<0.0001, Wilcoxon test, Figure S3), in line with the results on the association of genome size with the presence of a capsule locus. To control for the effect of the number of core genes on the observed association between HR and the presence of a capsule locus, we first made a principal component analysis on the correlations between the different measures of HR. The first axis explained 67% of the variance and was used in a generalized linear model to assess the association between the presence of the capsule locus and HR, when controlling for the number of core genes (Table S1). The results confirmed higher HR in C_sp_+ than in C_sp_- (P=0.02, general linear model, -GLM-).
