# Supplementary material for: Genetic exchanges are more frequent in bacteria encoding capsules
Source: PLoS Genet. 2018 Dec 21;14(12):e1007862. doi: 10.1371/journal.pgen.1007862 (PMC6322790; doi:10.1371/journal.pgen.1007862)
Supplement: S2 Text — (DOCX) [file pgen.1007862.s002.docx]

**Text S2. Controls for phylogenetic inertia.**

Most of the 127 species used in this study are very distant from all the others. They are part of 63 different genera. This means that phylogenetic inertia should be low and that it is going to be poorly estimated given the long internal branches in the tree. Nevertheless, we tested which traits had a significant Pagel's λ using a 16S rRNA tree on the Bacteria. We removed the four species from archaea from the analysis to avoid using a tree with poor resolution. We obtained values significant higher than zero for the pan-genome size (λ=0.55), and the first principal component of the PCA on the measures of HR (λ=0.25), whereas the value for HGT was at the borderline of statistical significance (λ=0.08, Table S2). We made a control by phylogeny for the three variables using generalized linear mixed models (GLMM) with the 16S phylogenetic tree linking all the bacterial species. The results confirm a significant association between the presence of the capsule locus and the size of the pan-genome (P=0. 0004), and the number of HGT events (P=0.009). The results for HR after phylogenetic control are at the borderline of the classical thresholds of statistical significance (P=0.078). It should be noted that Pagel's λ was also at the border of statistical significance (λ=0.08), which suggests that the relatively high P-value may just reflect low statistical power for this analysis.
