# Supplementary material for: Genetic exchanges are more frequent in bacteria encoding capsules
Source: PLoS Genet. 2018 Dec 21;14(12):e1007862. doi: 10.1371/journal.pgen.1007862 (PMC6322790; doi:10.1371/journal.pgen.1007862)
Supplement: S1 Fig — The tree was built using the 16S rRNA sequences of 122 bacterial species. From the inside to the outside: squares indicate the presence (full) or absence (empty) of capsule systems in all genomes of each species; rectangles represent the core (orange) and pan-genome (red) size, the number of homologous recombination events (blue) and total number of HGT events (green) (calculated as the sum of all gains and losses). Legends show minimum, median and maximum values. The circles along the branches are color-coded and proportional to bootstrap values. (DOCX) [file pgen.1007862.s003.docx]

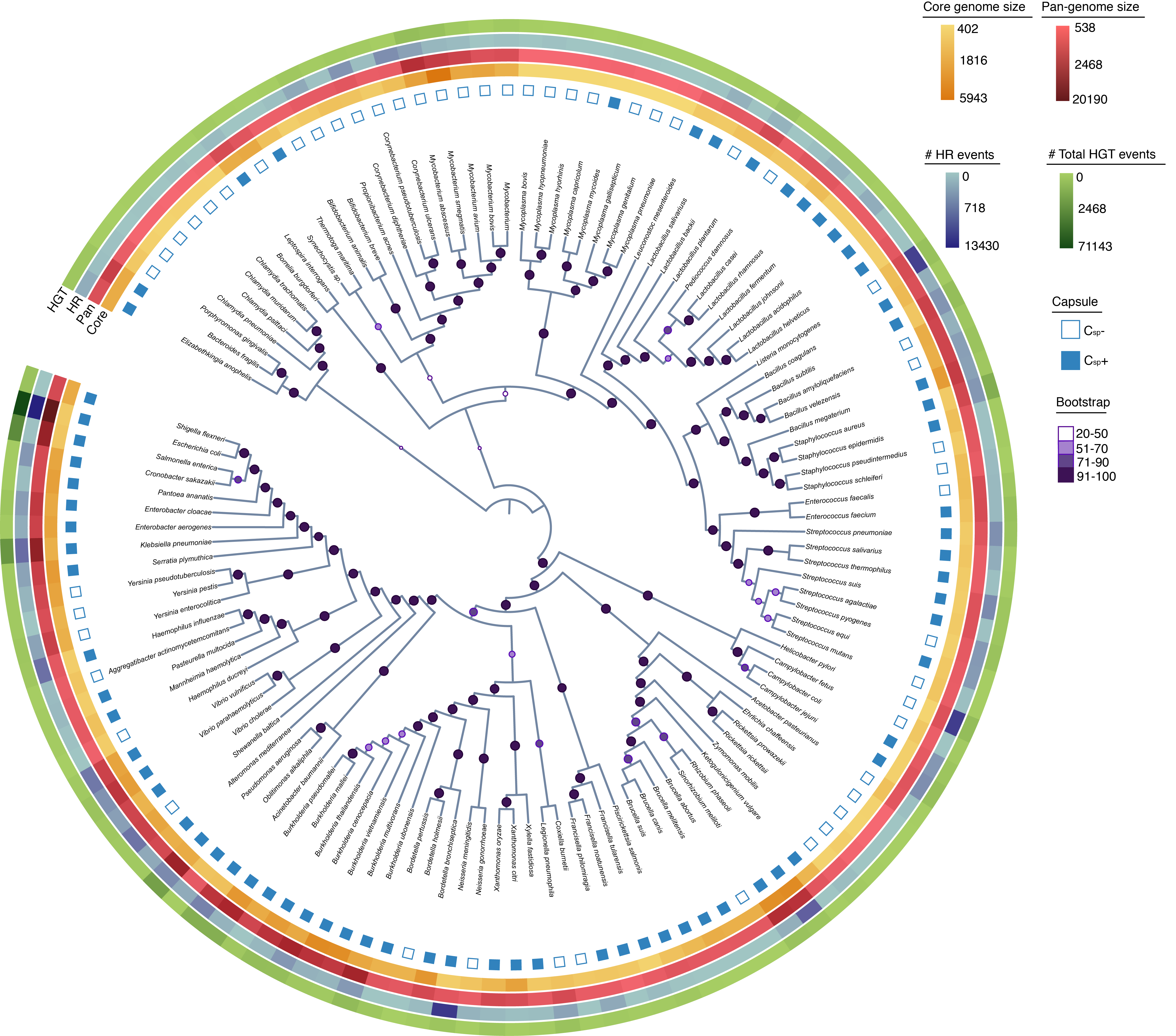


**Figure S1. Cladogram of analysed bacterial species.** The tree was built using the 16S rRNA sequences of 122 bacterial species. From the inside to the outside: squares indicate the presence (full) or absence (empty) of capsule systems in all genomes of each species; rectangles represent the core (orange) and pan-genome (red) size, the number of homologous recombination events (blue) and total number of HGT events (green) (calculated as the sum of all gains and losses). Legends show minimum, median and maximum values. The circles along the branches are color-coded and proportional to bootstrap values.
