# Supplementary material for: Genetic exchanges are more frequent in bacteria encoding capsules
Source: PLoS Genet. 2018 Dec 21;14(12):e1007862. doi: 10.1371/journal.pgen.1007862 (PMC6322790; doi:10.1371/journal.pgen.1007862)
Supplement: S2 Fig — A. Co-occurrence between the presence of capsule and competence system in our species database. Dashed line indicates the ratio of species encoding at least one capsule system in the database (51%). Pearson's χ2 test with Yates' continuity correction. N.S. = not significant. B. Number of recombination events as inferred by ClonalFrameML. C. Percentage of genes for which the null hypothesis of no homologous recombination was refuted by PHI program as measured by excess polymorphism (CHI), by phylogenetic incongruence (PHI) and neighbour similarity score (NSS). (DOCX) [file pgen.1007862.s004.docx]

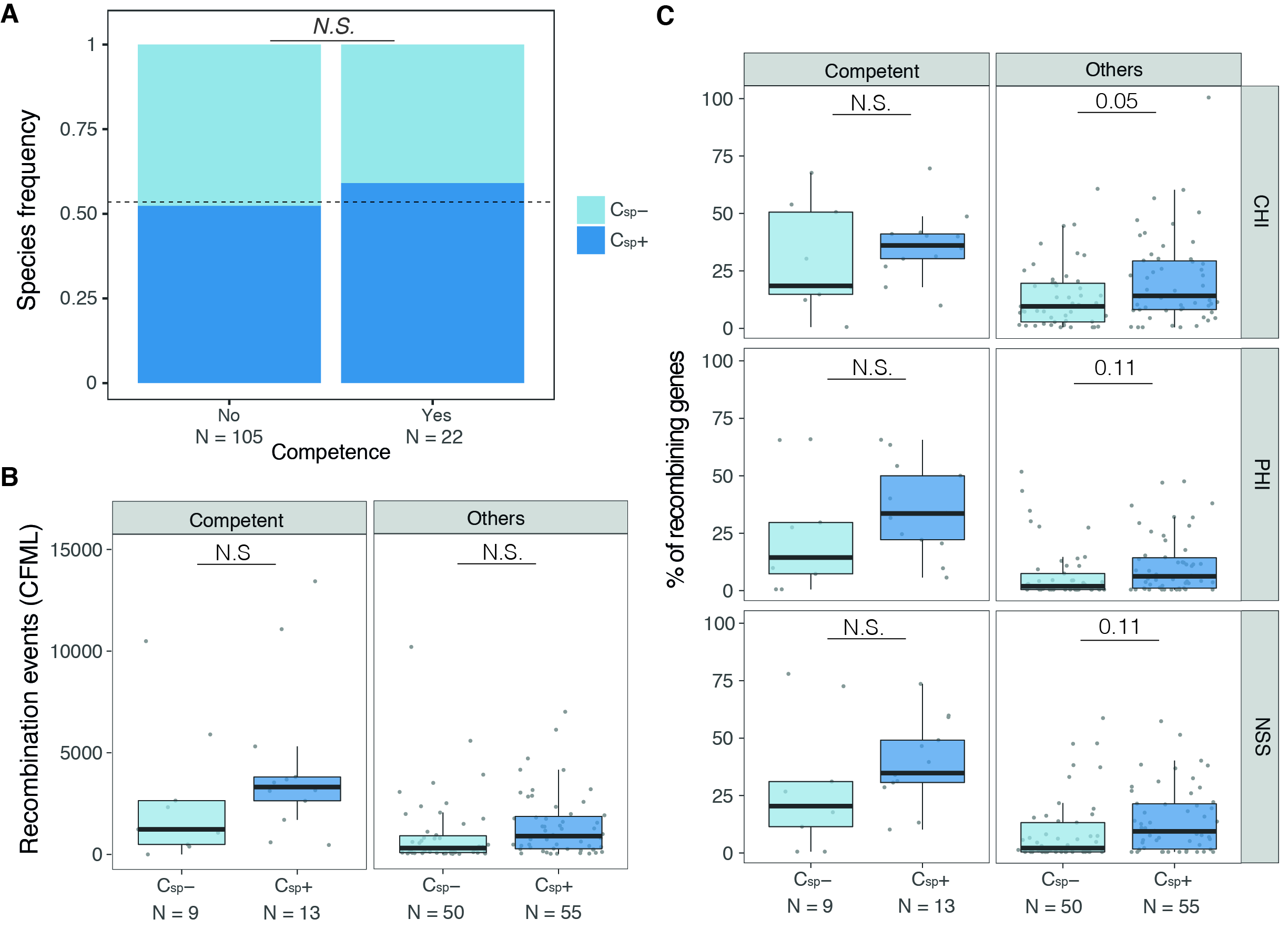


**Figure S2**. **Species recombination in function of capsule and competence for natural transformation**. **A.** Co-occurrence between the presence of capsule and competence system in our species database. Dashed line indicates the ratio of species encoding at least one capsule system in the database (51%). Pearson's χ^2^ test with Yates' continuity correction. *N.S.* = not significant. **B**. Number of recombination events as inferred by ClonalFrameML, GLM. **C**. Percentage of genes for which the null hypothesis of no homologous recombination was refuted by CHI, PHI, and NSS, GLM.
