# Supplementary material for: Genetic exchanges are more frequent in bacteria encoding capsules
Source: PLoS Genet. 2018 Dec 21;14(12):e1007862. doi: 10.1371/journal.pgen.1007862 (PMC6322790; doi:10.1371/journal.pgen.1007862)
Supplement: S3 Fig — Core genome size is expressed as the number of gene families present in all genomes of a given species (N = 127, ** P < 0.01, logistic regression controlled by genome size, S1 Table). (DOCX) [file pgen.1007862.s005.docx]

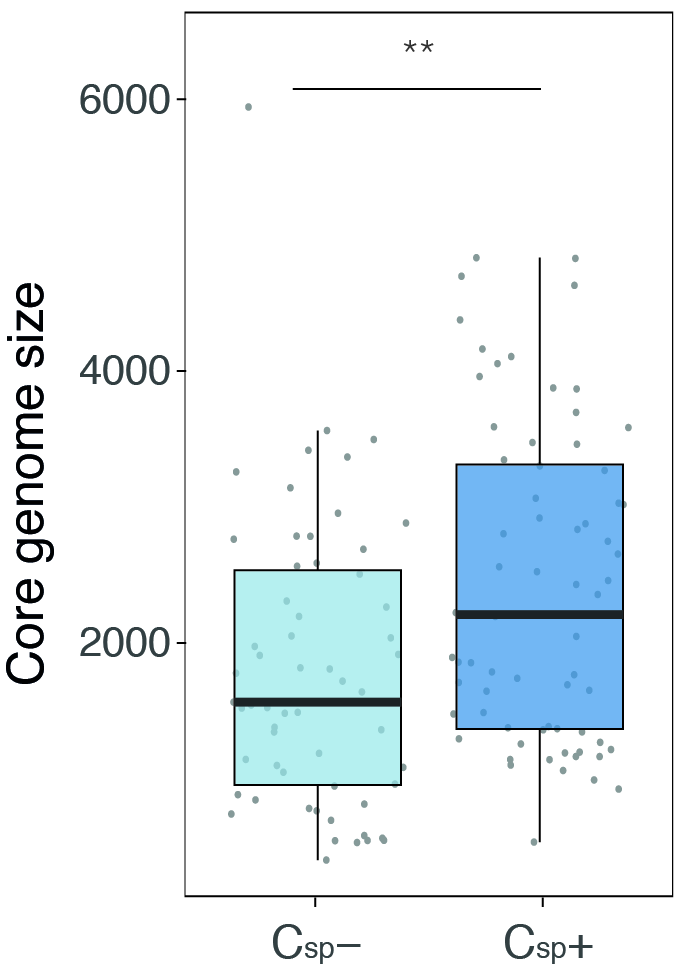


**Figure S3.** **Core genome size of species with (C_sp_+) and without capsule(C_sp_-).**  Core genome size is expressed as the number of gene families present in all genomes of a given species (*N*=127, ** P < 0.01, Wilcoxon test).
