# Supplementary material for: Genetic exchanges are more frequent in bacteria encoding capsules
Source: PLoS Genet. 2018 Dec 21;14(12):e1007862. doi: 10.1371/journal.pgen.1007862 (PMC6322790; doi:10.1371/journal.pgen.1007862)
Supplement: S4 Fig — All correlations are statistically significant, P < 0.001. (DOCX) [file pgen.1007862.s006.docx]

**Figure S4. Heatmap representing the Spearman's correlations between the different measures of recombination used in this study.** All correlations are statistically significant, P < 0.001.
