# Supplementary material for: Genetic exchanges are more frequent in bacteria encoding capsules
Source: PLoS Genet. 2018 Dec 21;14(12):e1007862. doi: 10.1371/journal.pgen.1007862 (PMC6322790; doi:10.1371/journal.pgen.1007862)
Supplement: S5 Fig — A. Percentage of genes for which the null hypothesis of no homologous recombination was rejected by the PhiPack program as measured by the tests: CHI, PHI, and NSS * P < 0.05, GLM. B. Number of recombination events as inferred by ClonalFrameML, ** P < 0.01, GLM. C. Comparisons of pan-genome size (expressed as the number of gene families) between species with and without capsule. D. Horizontal gene transfer as estimated by Wagner parsimony method (MRCA; most recent common ancestor). E. Horizontal gene transfer events as inferred by Count using birth-death models. * P < 0.05, GLM. Points represent individual species, and dispersion along the x-axis was done for visualization purposes. (DOCX) [file pgen.1007862.s007.docx]

**
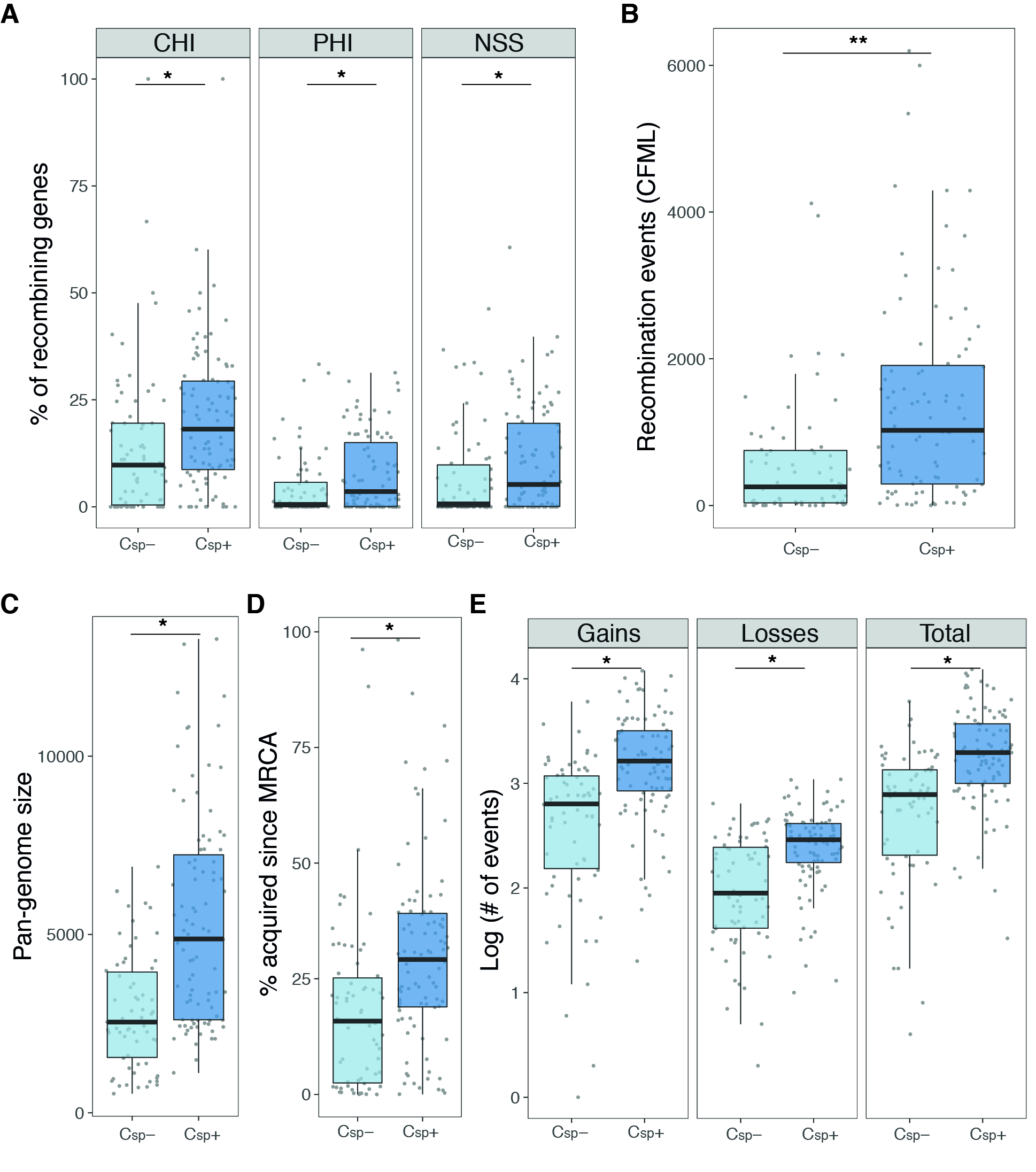
**

**Figure S5. Gene exchange in bacterial species is higher in species coding for capsules as calculated with the rarefied sets (five randomly chosen genomes per species).** **A**. Percentage of genes for which the null hypothesis of no homologous recombination was rejected by the PhiPack program as measured by the tests: CHI, PHI, and NSS * P < 0.05, GLM. **B**. Number of recombination events as inferred by ClonalFrameML, ** P < 0.01, GLM. **C**. Comparisons of pan-genome size (expressed as the number of gene families) between species with and without capsule. **D**. Horizontal gene transfer as estimated by Wagner parsimony method (MRCA; most recent common ancestor). **E.** Horizontal gene transfer events as inferred by Count using birth-death models. * P < 0.05, GLM. Points represent individual species, and dispersion along the x-axis was done for visualization purposes.
