# Supplementary material for: Genetic exchanges are more frequent in bacteria encoding capsules
Source: PLoS Genet. 2018 Dec 21;14(12):e1007862. doi: 10.1371/journal.pgen.1007862 (PMC6322790; doi:10.1371/journal.pgen.1007862)
Supplement: S7 Fig — Cumulative size of all prophages (A) and plasmids (B) per genome, log10-scale. Statistical test corresponds to a logistic regression controlled by genome size *** P <0.001 (DOCX) [file pgen.1007862.s009.docx]

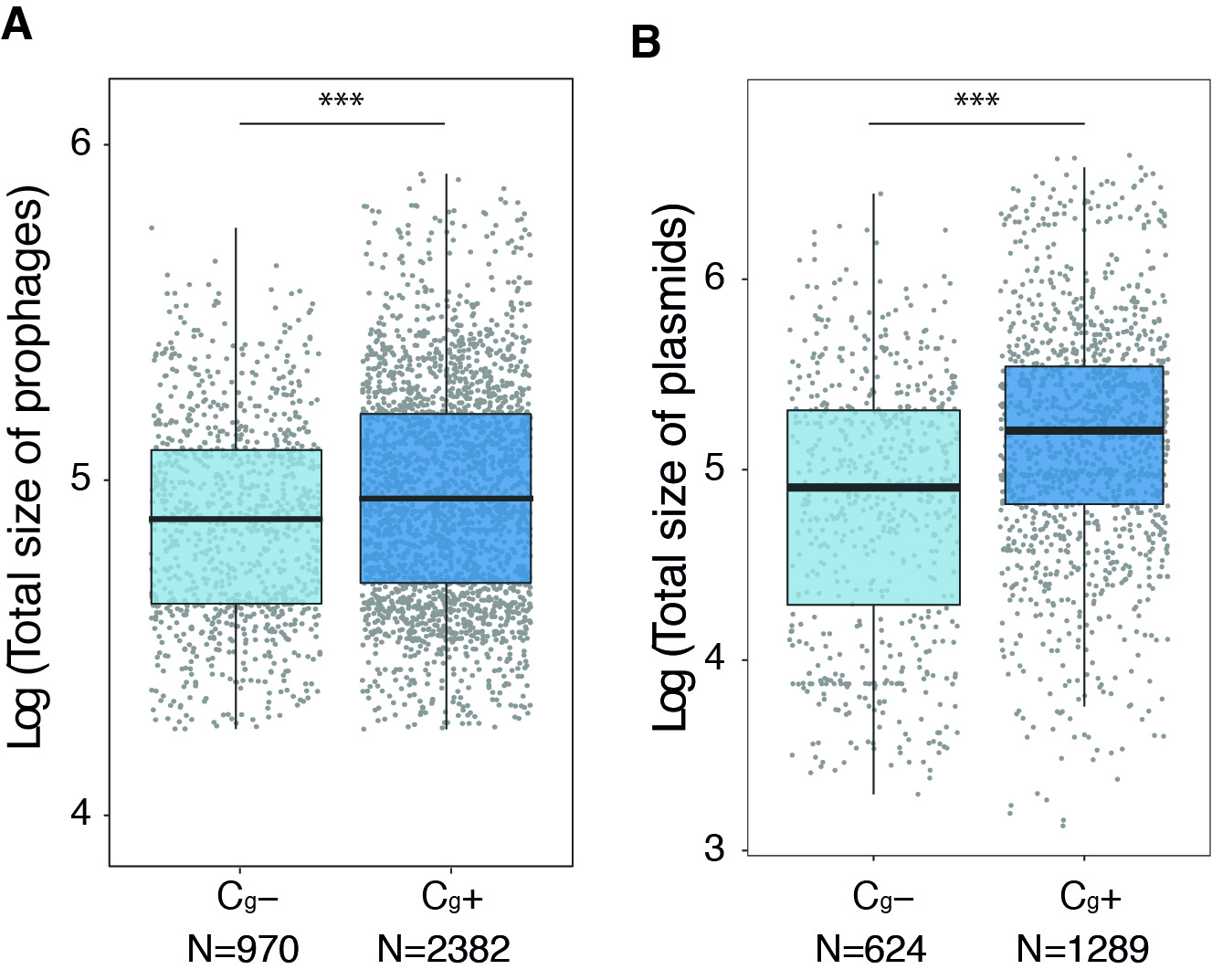


**Figure S7. Increased amount of foreign DNA in genomes coding for capsules.** Cumulative size of all prophages (**A**) and plasmids (**B**) per genome, log_10_-scale. Statistical test corresponds to a logistic regression controlled by genome size *** P <0.001
