# Supplementary material for: Genetic exchanges are more frequent in bacteria encoding capsules
Source: PLoS Genet. 2018 Dec 21;14(12):e1007862. doi: 10.1371/journal.pgen.1007862 (PMC6322790; doi:10.1371/journal.pgen.1007862)
Supplement: S8 Fig — Distribution of capsules in the chromosome and plasmids. Dashed line indicates the average across the whole dataset (~4%). (DOCX) [file pgen.1007862.s010.docx]

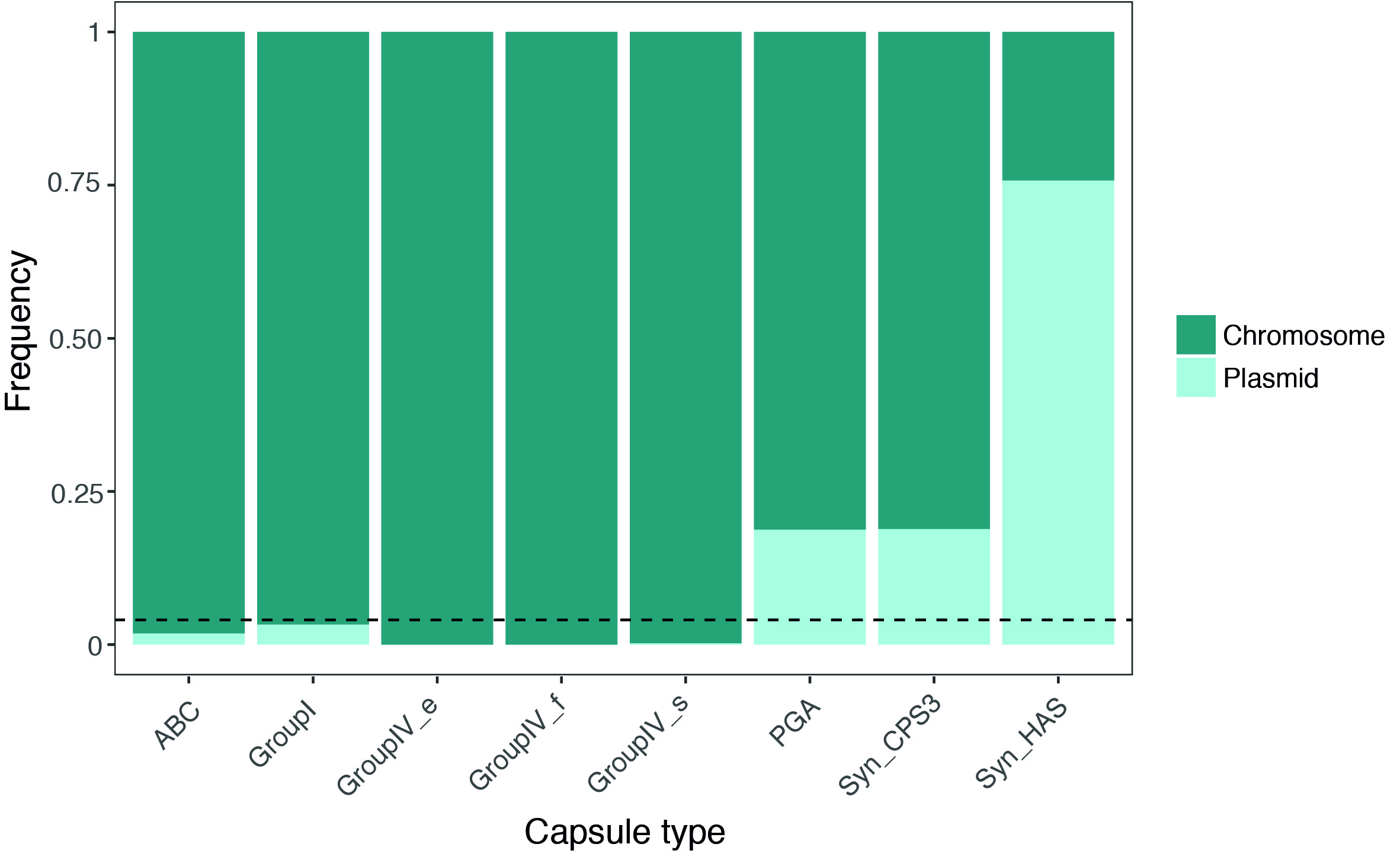


**Figure S8. Capsules encoded in plasmids.** Distribution of capsules in the chromosome and plasmids. Dashed line indicates the average across the whole dataset (~4%).
