# Supplementary material for: Genetic exchanges are more frequent in bacteria encoding capsules
Source: PLoS Genet. 2018 Dec 21;14(12):e1007862. doi: 10.1371/journal.pgen.1007862 (PMC6322790; doi:10.1371/journal.pgen.1007862)
Supplement: S9 Fig — CRISPR-Cas were identified as described in [96]. N.S. = not significant, Pearson’s χ2 test. (DOCX) [file pgen.1007862.s011.docx]

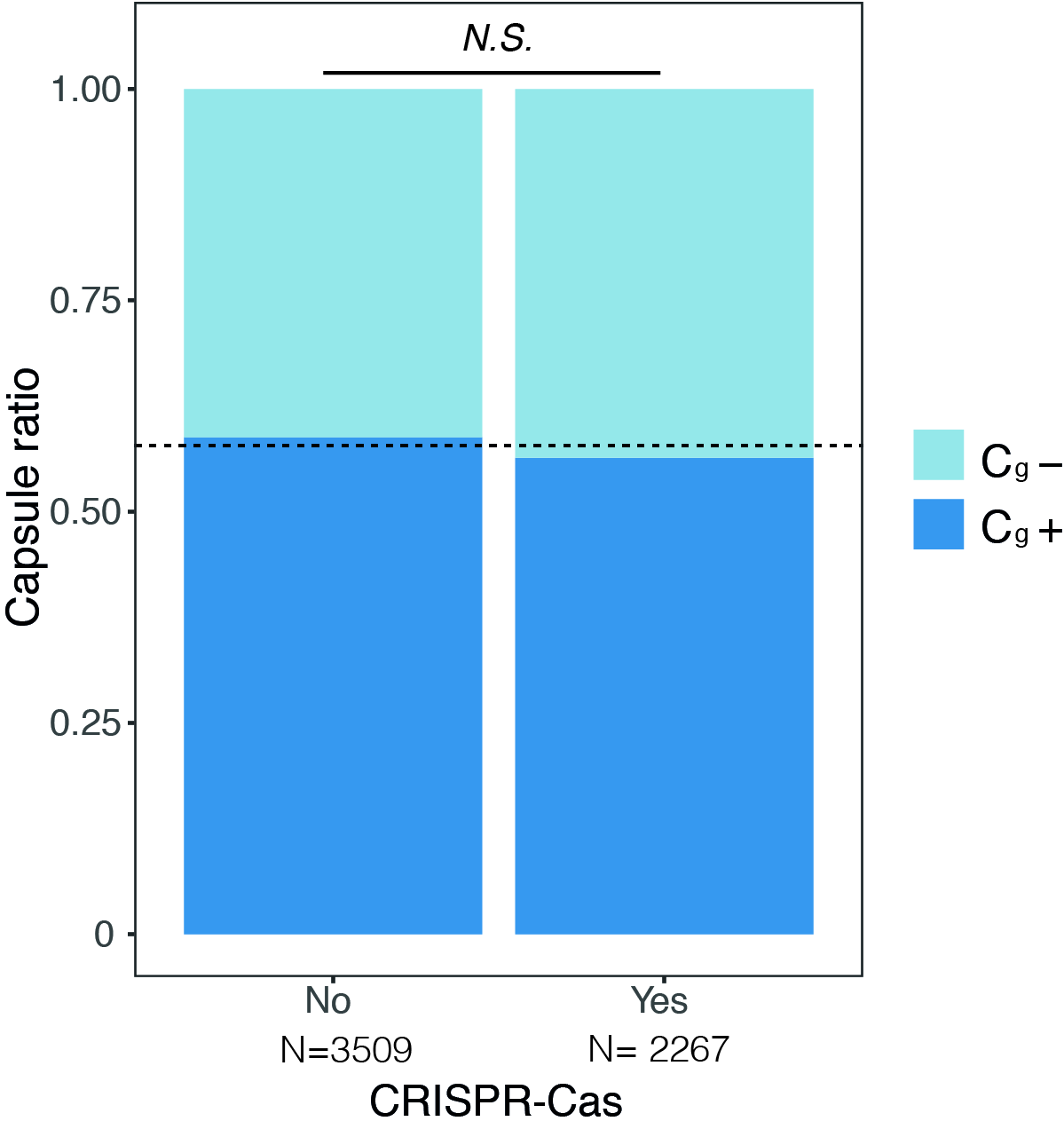


**Figure S9. Co-occurrence between capsule systems and CRISPR-Cas systems.** CRISPR-Cas were identified as described in (86). *N.S*.= not significant, Pearson’s $X$^2^ test.
