# Supplementary material for: Genetic exchanges are more frequent in bacteria encoding capsules
Source: PLoS Genet. 2018 Dec 21;14(12):e1007862. doi: 10.1371/journal.pgen.1007862 (PMC6322790; doi:10.1371/journal.pgen.1007862)
Supplement: S10 Fig — A. Number of systems of each capsule type detected in the dataset of 5576 genomes. Numbers on top of bars indicate percentage of each capsule type. B. Distribution of genomes encoding a capsule (Cg+) in each bacterial species for which we dispose of more than 4 genomes. We discarded species with more than 0.2 and less than 0.8 Cg+. (DOCX) [file pgen.1007862.s012.docx]

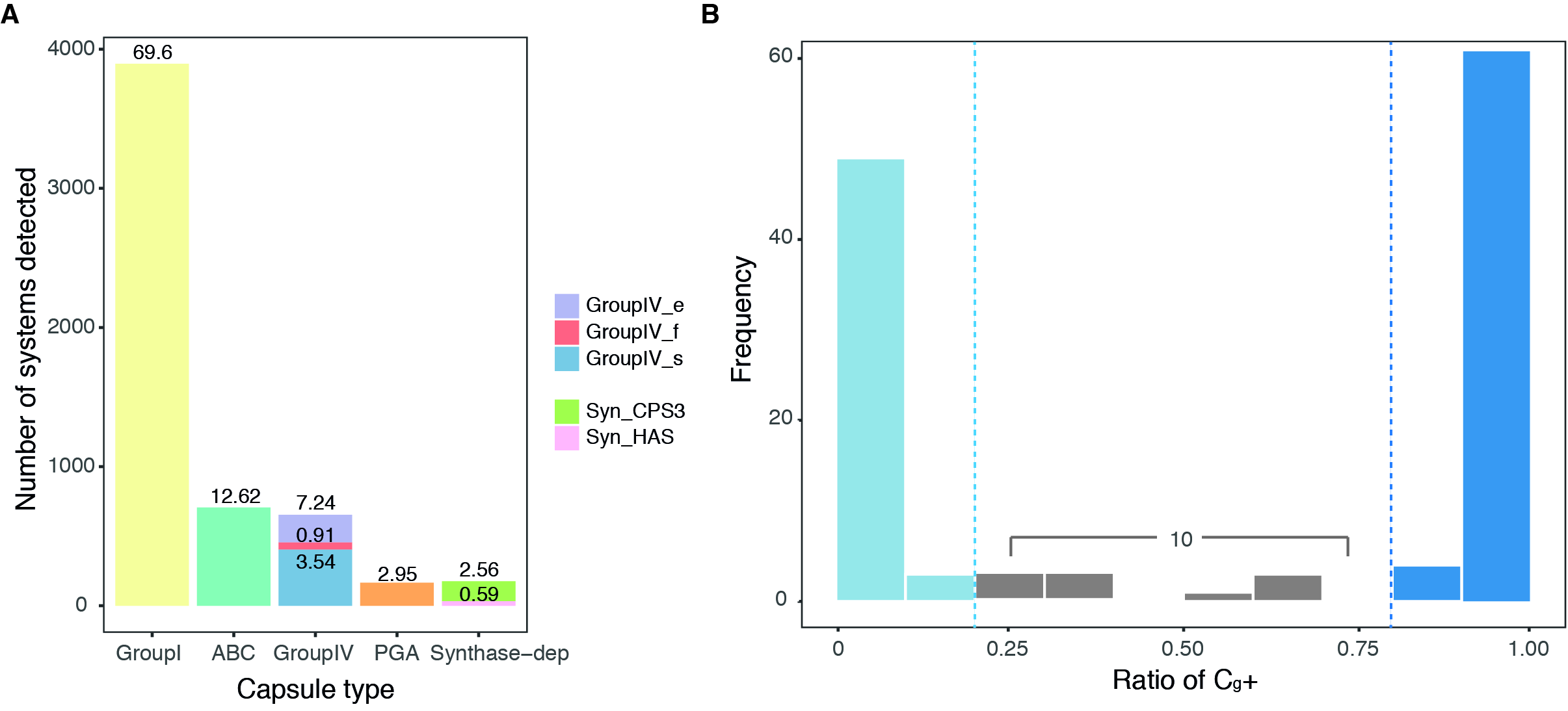


**Figure S10. Summary statistics of capsule systems detected in the database**. **A.** Number of systems of each capsule type detected in the dataset of 5576 genomes. Numbers on top of bars indicate percentage of each capsule type. **B.** Distribution of genomes encoding a capsule (C_g_+) in each bacterial species for which we dispose of more than 4 genomes. We discarded species with more than 0.2 and less than 0.8 C_g_+.
