# Supplementary figures and images for: Genetic exchanges are more frequent in bacteria encoding capsules
Source: PLoS Genet. 2018 Dec 21;14(12):e1007862. doi: 10.1371/journal.pgen.1007862 (PMC6322790; doi:10.1371/journal.pgen.1007862)

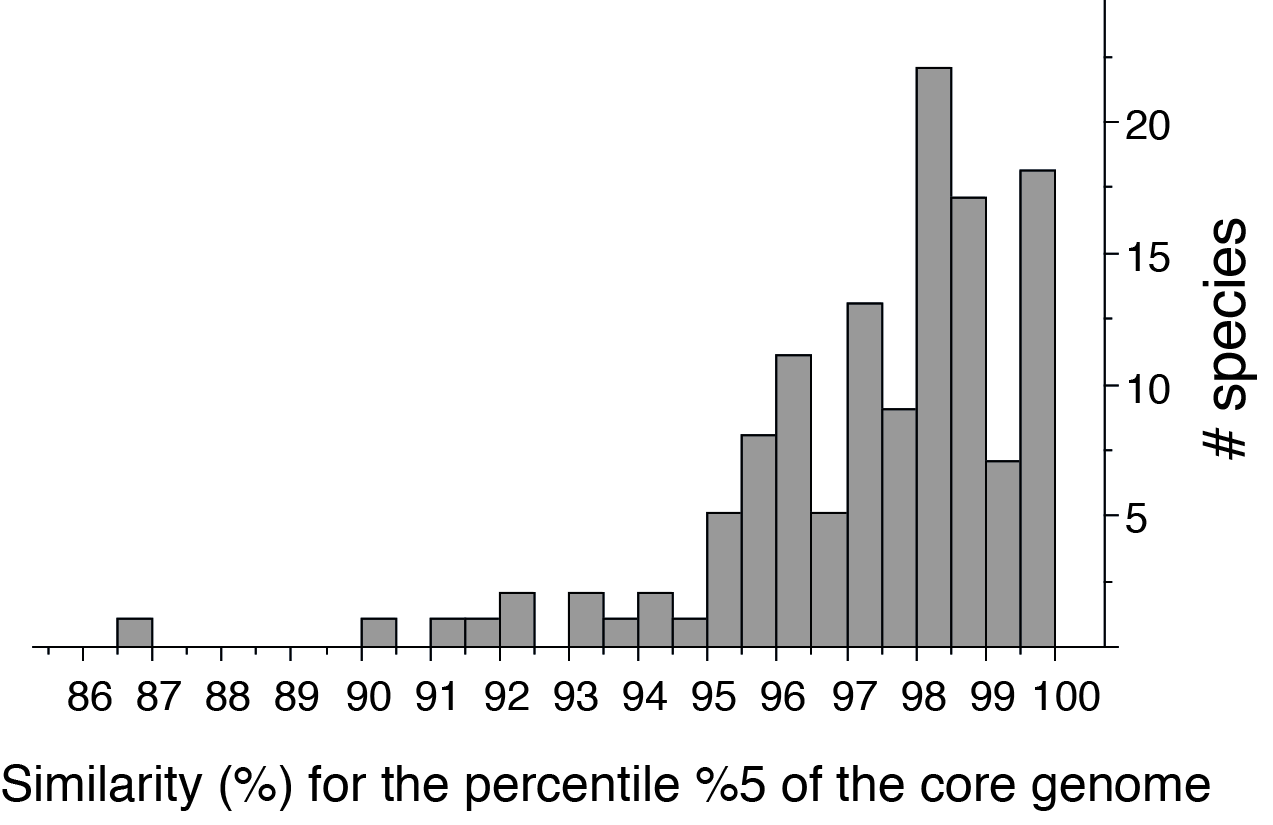


**Figure S11. Percentage of sequence similarity for the 5% percentile of the core genome.**

Supplement: S11 Fig — (DOCX) [file pgen.1007862.s013.docx]
