# Supplementary material for: Genetic exchanges are more frequent in bacteria encoding capsules
Source: PLoS Genet. 2018 Dec 21;14(12):e1007862. doi: 10.1371/journal.pgen.1007862 (PMC6322790; doi:10.1371/journal.pgen.1007862)
Supplement: S1 Table — We performed a logistic regression to control for genome size and other associated variables to the response trait. (DOCX) [file pgen.1007862.s014.docx]

**Table S1. Statistic details for rates of genetic exchange and genetic richness.** We used a generalized linear model (binomial distribution, logit link function) to control the key results for genome size (for the pan-genome and HGT) and core genome size (for homologous recombination).

| Y | X | control | P-value | test |
| --- | --- | --- | --- | --- |
| Capsule | Log(Pan-genome size) | Log(Genome size) | 0.0047 | GLM (Binomial,logit) |
| CapsulE | Log(HGT (gains+losses)) | Log(Genome size) | 0.0350 | GLM (Binomial,logit) |
| Capsule | HR (Prin1 Hrec) | Core genome size | 0.0248 | GLM (Binomial,logit) |
