# Supplementary material for: Genetic exchanges are more frequent in bacteria encoding capsules
Source: PLoS Genet. 2018 Dec 21;14(12):e1007862. doi: 10.1371/journal.pgen.1007862 (PMC6322790; doi:10.1371/journal.pgen.1007862)
Supplement: S2 Table — We estimated the phylogenetic inertia of several genetic transfer measures using Pagel’s λ included in the phytools package and a 16SrRNA phylogenetic tree. The null hypothesis is λ = 0 (no phylogenetic effect). (DOCX) [file pgen.1007862.s015.docx]

**Table S2. Phylogenetic inertia of gene transfer exchanges and capsule systems.** We estimated the phylogenetic inertia of the relevant variables using Pagel’s **λ** with the function included in the *phytools* package and a 16SrRNA phylogenetic tree. The null hypothesis is **λ** = 0 (no phylogenetic effect).

|  | Pagel’s λ | P value adjusted (Bonferroni) |
| --- | --- | --- |
| Log (Pan genome size) | 0.91 | < 0.001 |
| CHI | 0.18 | 0.15 |
| PHI | 0.31 | 0.1 |
| NSS | 0.33 | 0.06 |
| Recombination events CFML | 0.07 | 1 |
| HR (PC1) | 0.254 | 0.045 -> |
| Gains (HGT) | 0.08 | 0.57 |
| Losses (HGT) | 0.05 | 1 |
| HGT (gains+losses) | 0.079 | 0. 86 |
| Capsule | 0.49 | < 0.001 |

**Table S2 bis.**Phylogenetic regression results using the 16SrRNA phylogenetic tree.

| Y | X | P-value | test |
| --- | --- | --- | --- |
| Capsule | Log(Pan-genome size) | 0.00044 | binaryPGLMM |
| CapsulE | Log(HGT (gains+losses)) | 0.00936 | binaryPGLMM |
| Capsule | Prin1 Hrec | 0.07828 | binaryPGLMM |
