# Supplementary material for: Genetic exchanges are more frequent in bacteria encoding capsules
Source: PLoS Genet. 2018 Dec 21;14(12):e1007862. doi: 10.1371/journal.pgen.1007862 (PMC6322790; doi:10.1371/journal.pgen.1007862)
Supplement: S3 Table — We estimated the phylogenetic inertia of the presence of capsules and MGE in genomes using Pagel’s λ included in the phytools package and a 16SrRNA phylogenetic tree. The null hypothesis (λ = 0, no inertia) was always rejected. (DOCX) [file pgen.1007862.s016.docx]

**Table S3. Phylogenetic analysis of the distribution of MGEs and capsule systems.** We estimated the phylogenetic inertia of the presence of capsules and MGE in genomes using Pagel’s **λ** included in the *phytools* package and a 16SrRNA phylogenetic tree. The null hypothesis (**λ** =0, no inertia) was always rejected.

|  | **Pagel’s λ** | ***P*- value** |
| --- | --- | --- |
| Capsule | 0.98 | < 0.001 |
| Prophages | 0.69 | < 0.001 |
| Integrons | 0.85 | < 0.001 |
| Plasmid | 0.64 | < 0.001 |
