# Supplementary material for: Genetic exchanges are more frequent in bacteria encoding capsules
Source: PLoS Genet. 2018 Dec 21;14(12):e1007862. doi: 10.1371/journal.pgen.1007862 (PMC6322790; doi:10.1371/journal.pgen.1007862)
Supplement: S4 Table — To test for the co-occurrence of MGE and capsule in a genome, we used BayesTraitsv3 (see methods) to calculate the Bayes factor. Bayes Factors can be interpreted as follows: <2 weak evidence, >2 positive evidence, 5–10 strong evidence, and >10 very strong evidence. The lower evidence for Firmicutes may be associated with the smaller sample size. Genomes of Firmicutes (N = 1189) and Proteobacteria (N = 2897). (DOCX) [file pgen.1007862.s017.docx]

**Table S4**. **Phylogenetic corrections for association between MGEs and capsule systems**. To test for the co-occurrence of MGE and capsule in a genome, we used BayesTraitsv3 (see methods) to calculate the Bayes factor. Bayes Factors can be interpreted as follows: <2 weak evidence, >2 positive evidence, 5–10 strong evidence, and >10 very strong evidence. The lower evidence for Firmicutes may be associated with the smaller sample size. Genomes of Firmicutes (N=1189) and Proteobacteria (N=2897).

|  | **Bayes Factor**  **Proteobacteria** | **Bayes Factor**  **Firmicutes** |
| --- | --- | --- |
| Prophages | 85.5 ± 2.3 | 9.4 ± 0.3 |
| Integrons | 62.1 ± 2.0 | 3.0 ± 0.01 |
| Plasmids | 26.2 ± 2.0 | 5.6 ± 1.0 |

**Table S4 bis.** To test for the correlation between number of MGEs and the presence of a capsule system, we used the function *compar.gee,* a generalized estimating equation from the R package ape.

|  | **GEE**  **Proteobacteria** | **GEE**  **Firmicutes** |
| --- | --- | --- |
| Prophages | P = 0.04 | P < 0.001 |
| Integrons | P = 0.63 | - |
| Plasmids | P = 0.22 | P < 0.001 |
