# Supplementary material for: Genetic exchanges are more frequent in bacteria encoding capsules
Source: PLoS Genet. 2018 Dec 21;14(12):e1007862. doi: 10.1371/journal.pgen.1007862 (PMC6322790; doi:10.1371/journal.pgen.1007862)
Supplement: S5 Table — Only genomes with at least one MGE were taken into account. P-values corresponds to the test of difference between the mean number of MGEs in Cg+ and in Cg- (all corrected for genome size and phylogeny, see S3 Table for details). (DOCX) [file pgen.1007862.s018.docx]

**Table S5. Average number of mobile genetic elements per genome**. Only genomes with at least one MGE were taken into account. *P*-values corresponds to the test of difference between the mean number of MGEs in Cg+ and in Cg- (all corrected for genome size and phylogeny, see Table S3 for details).

|  | **Database** | **C_g_+** | **C_g_-** | ***P*-value** |
| --- | --- | --- | --- | --- |
| Prophages | 2.51 | 2.66 | 2.14 | < 0.001 |
| Integrons | 1.13 | 1.15 | 1.01 | 0.001 |
| Plasmids | 2.32 | 2.41 | 2.16 | 0.013 |
| Transposases | 31.99 | 32.05 | 31.84 | *N.S.* |
