# Supplementary material for: Genetic exchanges are more frequent in bacteria encoding capsules
Source: PLoS Genet. 2018 Dec 21;14(12):e1007862. doi: 10.1371/journal.pgen.1007862 (PMC6322790; doi:10.1371/journal.pgen.1007862)
Supplement: S6 Table — (DOCX) [file pgen.1007862.s019.docx]

**Table S6. List of plasmids with capsule systems.**

| **Capsule type** | **Replicon ID** | **Species** | **Plasmid name** | **Clade** | **Plasmid size** | **Transmission** | **Accession Number** |
| --- | --- | --- | --- | --- | --- | --- | --- |
| ABC | BUSP013.B.00001.P005 | *Burkholderia sp.* | p2_DNA | Betaproteobacteria | 309692 | Non-mobilizable | NC_021295 |
| ABC | CHSP002.B.00001.P002 | *Chelatococcus sp.* | pCO-6 | Alphaproteobacteria | 849688 | Non-mobilizable | NZ_CP012399 |
| ABC | CUME001.B.00001.P002 | *Cupriavidus metallidurans* | megaplasmid | Betaproteobacteria | 2580084 | Non-mobilizable | NC_007974 |
| ABC | CUSP005.B.00001.P003 | *Cupriavidus sp.* | unnamed1 | Betaproteobacteria | 544877 | Conjugative | NZ_CP017750 |
| ABC | ENAS001.B.00003.P002 | *Enterobacter asburiae* | pCAV1043-97 | Gammaproteobacteria | 96842 | Conjugative | NZ_CP011590 |
| ABC | NOSP007.B.00001.P003 | *Novosphingobium sp.* | Lpl | Alphaproteobacteria | 192103 | Conjugative | NC_015579 |
| ABC | OCTE001.B.00001.P002 | *Octadecabacter temperatus* | OSB_p1 | Alphaproteobacteria | 31560 | Non-mobilizable | NZ_CP012161 |
| ABC | PHGA002.B.00001.P007 | *Phaeobacter gallaeciensis* | pGal_F69 | Alphaproteobacteria | 68752 | Non-mobilizable | NC_023142 |
| ABC | PHIN001.B.00001.P004 | *Phaeobacter gallaeciensis* | pPGA2_71 | Alphaproteobacteria | 70384 | Non-mobilizable | NC_018422 |
| ABC | PHIN001.B.00002.P004 | *Phaeobacter inhibens* | pPGA1_65 | Alphaproteobacteria | 65245 | Non-mobilizable | NC_018288 |
| ABC | POSP002.B.00001.P002 | *Polaromonas sp.* | 1 | Betaproteobacteria | 360405 | Conjugative | NC_007949 |
| ABC | RHTR001.B.00001.P002 | *Rhizobium tropici* | pRtrCIAT899c | Alphaproteobacteria | 2083197 | Non-mobilizable | NC_020062 |
| ABC | RUMO001.B.00001.P005 | *Ruegeria mobilis* | unnamed4 | Alphaproteobacteria | 58318 | Non-mobilizable | NZ_CP015234 |
| GroupI | AGSP001.B.00001.P002 | *Agrobacterium sp.* | pBSY240_1 | Alphaproteobacteria | 323507 | Mobilizable | NZ_CP016500 |
| GroupI | AUSP001.B.00001.P002 | *Aureimonas sp.* | pAU20a | Alphaproteobacteria | 488888 | Non-mobilizable | NZ_CP006368 |
| GroupI | AZBR001.B.00001.P002 | *Azospirillum brasilense* | AbAZ39_p1 | Alphaproteobacteria | 1901707 | Non-mobilizable | NZ_CP007794 |
| GroupI | AZBR001.B.00001.P004 | *Azospirillum brasilense* | AbAZ39_p3 | Alphaproteobacteria | 686487 | Non-mobilizable | NZ_CP007796 |
| GroupI | AZBR001.B.00002.P002 | *Azospirillum brasilense* | ABSP7_p1 | Alphaproteobacteria | 1754523 | Non-mobilizable | NZ_CP012915 |
| GroupI | AZBR001.B.00002.P003 | *Azospirillum brasilense* | ABSP7_p2 | Alphaproteobacteria | 819020 | Non-mobilizable | NZ_CP012916 |
| GroupI | AZLI001.B.00002.P004 | *Azospirillum brasilense* | AZO_p3 | Alphaproteobacteria | 648491 | Non-mobilizable | NC_016623 |
| GroupI | AZSP001.B.00001.P004 | *Azospirillum sp.* | pAB510c_DNA | Alphaproteobacteria | 681723 | Non-mobilizable | NC_013857 |
| GroupI | BACE001.B.00015.P002 | *Bacillus cereus* | pBCX01 | Bacilli | 209381 | Non-mobilizable | NZ_CP009940 |
| GroupI | BACE001.B.00020.P002 | *Bacillus cereus* | pBC210 | Bacilli | 209255 | Non-mobilizable | NZ_CP009591 |
| GroupI | BACE001.B.00027.P002 | *Bacillus cereus* | pFORC13 | Bacilli | 259749 | Non-mobilizable | NZ_CP011146 |
| GroupI | BUGL001.B.00001.P003 | *Burkholderia gladioli* | bgla_4p | Betaproteobacteria | 403586 | Conjugative | NC_015383 |
| GroupI | BUSP013.B.00001.P003 | *Burkholderia sp.* | p1_DNA | Betaproteobacteria | 1275199 | Non-mobilizable | NC_021289 |
| GroupI | BUUB001.B.00002.P004 | *Burkholderia ubonensis* | pRF23 | Betaproteobacteria | 258679 | Conjugative | NZ_CP013369 |
| GroupI | CHSP002.B.00001.P002 | *Chelatococcus sp.* | pCO-6 | Alphaproteobacteria | 849688 | Non-mobilizable | NZ_CP012399 |
| GroupI | CUME001.B.00001.P002 | *Cupriavidus metallidurans* | NA | Betaproteobacteria | 2580084 | Non-mobilizable | NC_007974 |
| GroupI | DEPR001.B.00001.P004 | *Deinococcus proteolyticus* | pDEIPR03 | Deinococcus-Thermus | 132270 | Non-mobilizable | NC_015170 |
| GroupI | DEVU001.B.00001.P002 | *Desulfovibrio vulgaris* | pDVUL01 | Deltaproteobacteria | 198504 | Non-mobilizable | NC_008741 |
| GroupI | DEVU001.B.00003.P002 | *Desulfovibrio vulgaris* | pDEVAL01 | Deltaproteobacteria | 202305 | Non-mobilizable | NC_017311 |
| GroupI | DEVU001.B.00004.P002 | *Desulfovibrio vulgaris* | pDV | Deltaproteobacteria | 202301 | Non-mobilizable | NC_005863 |
| GroupI | DISH001.B.00001.P005 | *Dinoroseobacter shibae* | pDSHI04 | Alphaproteobacteria | 86208 | Non-mobilizable | NC_009958 |
| GroupI | ENAD001.B.00001.P002 | *Ensifer adhaerens* | pCasidaAA | Alphaproteobacteria | 1736943 | Non-mobilizable | NZ_CP015881 |
| GroupI | GRTU001.B.00001.P003 | *Granulicella tundricola* | pACIX902 | Acidobacteria | 300292 | Non-mobilizable | NC_015065 |
| GroupI | GRTU001.B.00001.P005 | *Granulicella tundricola* | pACIX904 | Acidobacteria | 115493 | Non-mobilizable | NC_015059 |
| GroupI | JASP003.B.00001.P002 | *Jannaschia sp.* | NA | Alphaproteobacteria | 86072 | Non-mobilizable | NC_007801 |
| GroupI | LABU001.B.00001.P002 | *Lactobacillus buchneri* | pLBUC01 | Bacilli | 52697 | Non-mobilizable | NC_015420 |
| GroupI | LABU001.B.00002.P002 | *Lactobacillus buchneri* | pCD034-3 | Bacilli | 56473 | Non-mobilizable | NC_018611 |
| GroupI | LAPL001.B.00004.P003 | *Lactobacillus plantarum* | pLP-ZJ102 | Bacilli | 39116 | Non-mobilizable | NC_021904 |
| GroupI | LAPL001.B.00006.P002 | *Lactobacillus plantarum* | Lp16H | Bacilli | 74078 | Conjugative | NC_021519 |
| GroupI | LAPL001.B.00011.P002 | *Lactobacillus plantarum* | pMK01 | Bacilli | 84759 | Conjugative | NZ_CP012657 |
| GroupI | LAPL001.B.00017.P003 | *Lactobacillus plantarum* | LZ227p1 | Bacilli | 74177 | Conjugative | NZ_CP015858 |
| GroupI | MAAD001.B.00001.P002 | *Marinobacter_adhaerens* | pHP-187 | Gammaproteobacteria | 187465 | Non-mobilizable | NC_017507 |
| GroupI | MECI001.B.00002.P002 | *Mesorhizobium ciceri* | pMc1192 | Alphaproteobacteria | 648231 | Conjugative | NZ_CP015063 |
| GroupI | MECI001.B.00003.P002 | *Mesorhizobium ciceri* | pMc1284 | Alphaproteobacteria | 553641 | Non-mobilizable | NZ_CP015065 |
| GroupI | MISP002.B.00001.P002 | *Mitsuaria sp.* | NA | Betaproteobacteria | 262673 | Non-mobilizable | NZ_CP011515 |
| GroupI | NORE001.B.00001.P002 | *Novosphingobium resinovorum* | pSA1 | Alphaproteobacteria | 1756808 | Conjugative | NZ_CP017076 |
| GroupI | PAPH004.B.00001.P003 | *Burkholderia phenoliruptrix* | pSYMBR3459 | Betaproteobacteria | 785419 | Conjugative | NC_018696 |
| GroupI | PASP017.B.00001.P003 | *Pantoea sp.* | pPAT9B02 | Gammaproteobacteria | 394054 | Conjugative | NC_014839 |
| GroupI | RASO001.B.00001.P002 | *Ralstonia solanacearum* | NA | Betaproteobacteria | 2094509 | Non-mobilizable | NC_003296 |
| GroupI | RASO001.B.00002.P002 | *Ralstonia solanacearum* | NA | Betaproteobacteria | 1949172 | Non-mobilizable | NC_017575 |
| GroupI | RASO001.B.00003.P002 | *Ralstonia solanacearum* | mpPSI07 | Betaproteobacteria | 2085000 | Non-mobilizable | NC_014310 |
| GroupI | RASO001.B.00004.P002 | *Ralstonia solanacearum* | NA | Betaproteobacteria | 1999545 | Non-mobilizable | NZ_CP012688 |
| GroupI | RASO001.B.00005.P002 | *Ralstonia solanacearum* | NA | Betaproteobacteria | 1932001 | Non-mobilizable | NZ_CP012940 |
| GroupI | RASO001.B.00006.P002 | *Ralstonia solanacearum* | NA | Betaproteobacteria | 2069788 | Non-mobilizable | NZ_CP012944 |
| GroupI | RASO001.B.00007.P002 | *Ralstonia solanacearum* | NA | Betaproteobacteria | 1907366 | Non-mobilizable | NZ_CP015851 |
| GroupI | RASO001.B.00008.P002 | *Ralstonia solanacearum* | unnamed1 | Betaproteobacteria | 2145732 | Non-mobilizable | NZ_CP016905 |
| GroupI | RHET001.B.00001.P002 | *Rhizobium etli* | pC | Alphaproteobacteria | 1091523 | Conjugative | NC_010997 |
| GroupI | RHET001.B.00004.P002 | *Rhizobium etli* | pRetIE4771e | Alphaproteobacteria | 744104 | Conjugative | NZ_CP006991 |
| GroupI | RHET001.B.00005.P002 | *Rhizobium etli* | pRetIE4803d | Alphaproteobacteria | 742914 | Conjugative | NZ_CP007645 |
| GroupI | RHGA001.B.00001.P002 | *Rhizobium gallicum* | pRgalR602c | Alphaproteobacteria | 2466951 | Non-mobilizable | NZ_CP006880 |
| GroupI | RHLE001.B.00001.P005 | *Rhizobium leguminosarum* | pRL9 | Alphaproteobacteria | 352782 | Non-mobilizable | NC_008379 |
| GroupI | RHLE001.B.00002.P005 | *Rhizobium leguminosarum* | pRLG204 | Alphaproteobacteria | 257956 | Non-mobilizable | NC_011371 |
| GroupI | RHLE001.B.00003.P005 | *Rhizobium leguminosarum* | pR132504 | Alphaproteobacteria | 350312 | Non-mobilizable | NC_012852 |
| GroupI | RHLE001.B.00004.P006 | *Rhizobium leguminosarum* | NA | Alphaproteobacteria | 259408 | Non-mobilizable | NZ_CP007050 |
| GroupI | RHLE001.B.00005.P002 | *Rhizobium leguminosarum* | NA | Alphaproteobacteria | 1563772 | Conjugative | NZ_CP007068 |
| GroupI | RHLE001.B.00006.P006 | *Rhizobium leguminosarum* | unnamed5 | Alphaproteobacteria | 274981 | Non-mobilizable | NZ_CP016293 |
| GroupI | RHPH001.B.00001.P002 | *Rhizobium phaseoli* | pRphaN261d | Alphaproteobacteria | 1074794 | Conjugative | NZ_CP013584 |
| GroupI | RHPH001.B.00002.P002 | *Rhizobium phaseoli* | pRphaN831d | Alphaproteobacteria | 1165895 | Non-mobilizable | NZ_CP013567 |
| GroupI | RHPH001.B.00003.P002 | *Rhizobium phaseoli* | pRphaN161d | Alphaproteobacteria | 1048055 | Conjugative | NZ_CP013589 |
| GroupI | RHPH001.B.00004.P002 | *Rhizobium phaseoli* | pRphaR744d | Alphaproteobacteria | 1195489 | Conjugative | NZ_CP013526 |
| GroupI | RHPH001.B.00004.P005 | *Rhizobium phaseoli* | pRphaR744a | Alphaproteobacteria | 256181 | Non-mobilizable | NZ_CP013523 |
| GroupI | RHPH001.B.00005.P002 | *Rhizobium phaseoli* | pRphaR630d | Alphaproteobacteria | 1144409 | Conjugative | NZ_CP013541 |
| GroupI | RHPH001.B.00006.P002 | *Rhizobium phaseoli* | pRphaR723d | Alphaproteobacteria | 1074795 | Conjugative | NZ_CP013531 |
| GroupI | RHPH001.B.00007.P002 | *Rhizobium phaseoli* | pRphaR620d | Alphaproteobacteria | 1080727 | Conjugative | NZ_CP013546 |
| GroupI | RHPH001.B.00008.P002 | *Rhizobium phaseoli* | pRphaN931d | Alphaproteobacteria | 1165895 | Non-mobilizable | NZ_CP013556 |
| GroupI | RHPH001.B.00009.P002 | *Rhizobium phaseoli* | pRphaN671e | Alphaproteobacteria | 1189373 | Conjugative | NZ_CP013579 |
| GroupI | RHPH001.B.00010.P002 | *Rhizobium phaseoli* | pRphaR650d | Alphaproteobacteria | 990934 | Non-mobilizable | NZ_CP013536 |
| GroupI | RHPH001.B.00011.P002 | *Rhizobium phaseoli* | pRphaN771e | Alphaproteobacteria | 1189373 | Conjugative | NZ_CP013573 |
| GroupI | RHPH001.B.00012.P002 | *Rhizobium phaseoli* | pRphaN841e | Alphaproteobacteria | 1201422 | Conjugative | NZ_CP013562 |
| GroupI | RHPH001.B.00013.P002 | *Rhizobium phaseoli* | pRetR611d | Alphaproteobacteria | 990934 | Non-mobilizable | NZ_CP013551 |
| GroupI | RHSP002.B.00002.P002 | *Rhodobacter sphaeroides* | pRSPA01 | Alphaproteobacteria | 877879 | Non-mobilizable | NC_009429 |
| GroupI | RHSP002.B.00002.P003 | *Rhodobacter sphaeroides* | pRSPA02 | Alphaproteobacteria | 289489 | Non-mobilizable | NC_009430 |
| GroupI | RHSP015.B.00001.P005 | *Rhizobium sp.* | pRspN324b | Alphaproteobacteria | 407833 | Non-mobilizable | NZ_CP013632 |
| GroupI | RHSU001.B.00001.P004 | *Rhodovulum_sulfidophilum* | Plasmid3_DNA | Alphaproteobacteria | 60897 | Non-mobilizable | NZ_AP014803 |
| GroupI | RHTR001.B.00001.P002 | *Rhizobium tropici* | pRtrCIAT899c | Alphaproteobacteria | 2083197 | Non-mobilizable | NC_020062 |
| GroupI | RODE002.B.00001.P003 | *Roseobacter_denitrificans* | pTB2 | Alphaproteobacteria | 69269 | Non-mobilizable | NC_008387 |
| GroupI | ROLI001.B.00001.P004 | *Roseobacter_litoralis* | pRLO149_63 | Alphaproteobacteria | 63532 | Non-mobilizable | NC_015729 |
| GroupI | SHSP005.B.00001.P003 | *Shinella sp.* | pShin-02 | Alphaproteobacteria | 445803 | Non-mobilizable | NZ_CP015738 |
| GroupI | SHSP005.B.00001.P004 | *Shinella sp.* | pShin-03 | Alphaproteobacteria | 409126 | Non-mobilizable | NZ_CP015739 |
| GroupI | SIFR001.B.00001.P002 | *Sinorhizobium fredii* | pNGR234b | Alphaproteobacteria | 2430033 | Conjugative | NC_012586 |
| GroupI | SIME001.B.00001.P002 | *Sinorhizobium medicae* | pSMED01 | Alphaproteobacteria | 1570951 | Non-mobilizable | NC_009620 |
| GroupI | SIME002.B.00001.P002 | *Sinorhizobium meliloti* | pSymB | Alphaproteobacteria | 1683333 | Non-mobilizable | NC_003078 |
| GroupI | SIME002.B.00002.P002 | *Sinorhizobium meliloti* | pSINMEB02 | Alphaproteobacteria | 1690594 | Non-mobilizable | NC_017323 |
| GroupI | SIME002.B.00004.P003 | *Sinorhizobium meliloti* | pSmeSM11d | Alphaproteobacteria | 1632395 | Non-mobilizable | NC_017326 |
| GroupI | SIME002.B.00005.P002 | *Sinorhizobium meliloti* | pSYMB | Alphaproteobacteria | 1664896 | Non-mobilizable | NC_018701 |
| GroupI | SIME002.B.00006.P002 | *Sinorhizobium meliloti* | pRmeGR4d | Alphaproteobacteria | 1701381 | Non-mobilizable | NC_019849 |
| GroupI | SIME002.B.00007.P002 | *Sinorhizobium meliloti* | pSymB | Alphaproteobacteria | 1683348 | Non-mobilizable | NC_020560 |
| GroupI | SIME002.B.00008.P002 | *Sinorhizobium meliloti* | pSymB | Alphaproteobacteria | 1617037 | Non-mobilizable | NZ_CP009146 |
| GroupI | SISP001.B.00001.P002 | *Sinorhizobium sp.* | pBSY16_1 | Alphaproteobacteria | 2220160 | Non-mobilizable | NZ_CP016452 |
| GroupI | SPHE001.B.00001.P002 | *Sphingomonas sp.* | NA | Alphaproteobacteria | 36853 | Non-mobilizable | NZ_CP010837 |
| GroupI | SPSP002.B.00001.P003 | *Sphingobium sp.* | pTK1 | Alphaproteobacteria | 520614 | Conjugative | NZ_CP005085 |
| GroupI | SPSP012.B.00001.P002 | *Sphingobium sp.* | 3pYBL2-3 | Alphaproteobacteria | 322226 | Mobilizable | NZ_CP010957 |
| GroupI | SPTA001.B.00001.P002 | *Sphingomonas taxi* | STP1 | Alphaproteobacteria | 163311 | Non-mobilizable | NZ_CP009572 |
| GroupI | YASP001.B.00001.P004 | *Yangia sp.* | unnamed3 | Alphaproteobacteria | 280527 | Non-mobilizable | NZ_CP014599 |
| GroupI | YASP001.B.00001.P005 | *Yangia sp.* | unnamed2 | Alphaproteobacteria | 274486 | Non-mobilizable | NZ_CP014598 |
| PGA | BAAN002.B.00003.P003 | *Bacillus anthracis* | pXO2 | Bacilli | 94830 | Non-mobilizable | NC_007323 |
| PGA | BAAN002.B.00004.P003 | *Bacillus anthracis* | pX02 | Bacilli | 94875 | Non-mobilizable | NC_012577 |
| PGA | BAAN002.B.00005.P003 | *Bacillus anthracis* | pXO2 | Bacilli | 94830 | Non-mobilizable | NC_012655 |
| PGA | BAAN002.B.00006.P003 | *Bacillus anthracis* | BAP2 | Bacilli | 94824 | Non-mobilizable | NC_017727 |
| PGA | BAAN002.B.00008.P003 | *Bacillus anthracis* | pXO2 | Bacilli | 94758 | Non-mobilizable | NZ_CP006744 |
| PGA | BAAN002.B.00009.P003 | *Bacillus anthracis* | pX02 | Bacilli | 94732 | Non-mobilizable | NZ_CP008848 |
| PGA | BAAN002.B.00010.P002 | *Bacillus anthracis* | pXO2 | Bacilli | 94756 | Non-mobilizable | NZ_CP007617 |
| PGA | BAAN002.B.00011.P003 | *Bacillus anthracis* | NA | Bacilli | 94889 | Non-mobilizable | NZ_CP007664 |
| PGA | BAAN002.B.00012.P003 | *Bacillus anthracis* | pXO2 | Bacilli | 94838 | Non-mobilizable | NZ_CP010794 |
| PGA | BAAN002.B.00014.P003 | *Bacillus anthracis* | 2 | Bacilli | 94892 | Non-mobilizable | NZ_CP009326 |
| PGA | BAAN002.B.00015.P003 | *Bacillus anthracis* | 2 | Bacilli | 94803 | Non-mobilizable | NZ_CP009329 |
| PGA | BAAN002.B.00016.P003 | *Bacillus anthracis* | 2 | Bacilli | 94797 | Non-mobilizable | NZ_CP009339 |
| PGA | BAAN002.B.00017.P003 | *Bacillus anthracis* | pXO2 | Bacilli | 94884 | Non-mobilizable | NZ_CP009462 |
| PGA | BAAN002.B.00018.P002 | *Bacillus anthracis* | pXO2 | Bacilli | 94721 | Non-mobilizable | NZ_CP009475 |
| PGA | BAAN002.B.00020.P003 | *Bacillus anthracis* | pXO2 | Bacilli | 94807 | Non-mobilizable | NZ_CP009542 |
| PGA | BAAN002.B.00021.P003 | *Bacillus anthracis* | pXO2 | Bacilli | 94767 | Non-mobilizable | NZ_CP009698 |
| PGA | BAAN002.B.00022.P003 | *Bacillus anthracis* | pXO2 | Bacilli | 94740 | Non-mobilizable | NZ_CP009695 |
| PGA | BAAN002.B.00024.P003 | *Bacillus anthracis* | pXO2 | Bacilli | 94747 | Non-mobilizable | NZ_CP009900 |
| PGA | BAAN002.B.00025.P003 | *Bacillus anthracis* | plasmid2 | Bacilli | 94831 | Non-mobilizable | NZ_CP009979 |
| PGA | BAAN002.B.00026.P003 | *Bacillus anthracis* | 2 | Bacilli | 94810 | Non-mobilizable | NZ_CP010320 |
| PGA | BAAN002.B.00027.P003 | *Bacillus anthracis* | NA | Bacilli | 94807 | Non-mobilizable | NZ_CP009314 |
| PGA | BAAN002.B.00028.P003 | *Bacillus anthracis* | pXO2 | Bacilli | 95135 | Non-mobilizable | NZ_CP010854 |
| PGA | BAAN002.B.00029.P003 | *Bacillus anthracis* | pXO2 | Bacilli | 94830 | Non-mobilizable | NZ_CP014178 |
| PGA | BAAN002.B.00030.P003 | *Bacillus anthracis* | pXO2 | Bacilli | 94830 | Non-mobilizable | NZ_CP015778 |
| PGA | BACE001.B.00009.P002 | *Bacillus cereus* | p03BB102_179 | Bacilli | 179680 | Non-mobilizable | NC_012473 |
| PGA | BACE001.B.00010.P003 | *Bacillus cereus* | pCI-XO2 | Bacilli | 94469 | Non-mobilizable | NC_014332 |
| PGA | BACE001.B.00017.P002 | *Bacillus cereus* | NA | Bacilli | 179680 | Non-mobilizable | NZ_CP009317 |
| PGA | BACE001.B.00022.P003 | *Bacillus cereus* | pBFI_2 | Bacilli | 238933 | Non-mobilizable | NZ_CP009636 |
| PGA | BATH001.B.00033.P002 | *Bacillus thuringiensis* | pBMB267 | Bacilli | 267609 | Non-mobilizable | NZ_CP015177 |
| PGA | CHTH003.B.00001.P002 | *Chroococcidiopsis thermalis* | pCHRO.01 | Cyanobacteria | 370830 | Conjugative | NC_019699 |
| PGA | COGL001.B.00001.P002 | *Corynebacterium glyciniphilum* | pCgly1 | Actinobacteria | 58432 | Non-mobilizable | NZ_CP006843 |
| Syn_CPS3 | MEEV001.B.00001.P002 | *Methanohalobium evestigatum* | pMETEV01 | Euryarchaeota | 163915 | Non-mobilizable | NC_014254 |
| Syn_CPS3 | SARU001.B.00002.P002 | *Salinibacter ruber* | pSR84 | Bacteroidetes | 84340 | Non-mobilizable | NC_014157 |
| Syn_HAS | BAAN002.B.00003.P002 | *Bacillus anthracis* | pXO1 | Bacilli | 181677 | Non-mobilizable | NC_007322 |
| Syn_HAS | BAAN002.B.00004.P002 | *Bacillus anthracis* | pX01 | Bacilli | 181773 | Non-mobilizable | NC_012579 |
| Syn_HAS | BAAN002.B.00005.P002 | *Bacillus anthracis* | pXO1 | Bacilli | 181677 | Non-mobilizable | NC_012656 |
| Syn_HAS | BAAN002.B.00006.P002 | *Bacillus anthracis* | BAP1 | Bacilli | 181700 | Non-mobilizable | NC_017726 |
| Syn_HAS | BAAN002.B.00007.P002 | *Bacillus anthracis* | pXO1 | Bacilli | 181763 | Non-mobilizable | NZ_CP001975 |
| Syn_HAS | BAAN002.B.00008.P002 | *Bacillus anthracis* | pXO1 | Bacilli | 181793 | Non-mobilizable | NZ_CP006743 |
| Syn_HAS | BAAN002.B.00009.P002 | *Bacillus anthracis* | pX01 | Bacilli | 181894 | Non-mobilizable | NZ_CP008847 |
| Syn_HAS | BAAN002.B.00012.P002 | *Bacillus anthracis* | pXO1 | Bacilli | 181677 | Non-mobilizable | NZ_CP010793 |
| Syn_HAS | BAAN002.B.00013.P002 | *Bacillus anthracis* | 1 | Bacilli | 181754 | Non-mobilizable | NZ_CP009324 |
| Syn_HAS | BAAN002.B.00016.P002 | *Bacillus anthracis* | 1 | Bacilli | 181369 | Non-mobilizable | NZ_CP009340 |
| Syn_HAS | BAAN002.B.00017.P002 | *Bacillus anthracis* | pXO1 | Bacilli | 180693 | Non-mobilizable | NZ_CP009463 |
| Syn_HAS | BAAN002.B.00019.P002 | *Bacillus anthracis* | pXO1 | Bacilli | 181624 | Non-mobilizable | NZ_CP009540 |
| Syn_HAS | BAAN002.B.00020.P002 | *Bacillus anthracis* | pXO1 | Bacilli | 181707 | Non-mobilizable | NZ_CP009543 |
| Syn_HAS | BAAN002.B.00021.P002 | *Bacillus anthracis* | pXO1 | Bacilli | 181892 | Non-mobilizable | NZ_CP009699 |
| Syn_HAS | BAAN002.B.00022.P002 | *Bacillus anthracis* | pXO1 | Bacilli | 181920 | Non-mobilizable | NZ_CP009696 |
| Syn_HAS | BAAN002.B.00023.P002 | *Bacillus anthracis* | pXO1 | Bacilli | 181710 | Non-mobilizable | NZ_CP009597 |
| Syn_HAS | BAAN002.B.00025.P002 | *Bacillus anthracis* | plasmid1 | Bacilli | 181674 | Non-mobilizable | NZ_CP009980 |
| Syn_HAS | BAAN002.B.00027.P002 | *Bacillus anthracis* | NA | Bacilli | 181760 | Non-mobilizable | NZ_CP009316 |
| Syn_HAS | BAAN002.B.00028.P002 | *Bacillus anthracis* | pXO1 | Bacilli | 181663 | Non-mobilizable | NZ_CP010853 |
| Syn_HAS | BAAN002.B.00029.P002 | *Bacillus anthracis* | pXO1 | Bacilli | 181677 | Non-mobilizable | NZ_CP014177 |
| Syn_HAS | BAAN002.B.00030.P002 | *Bacillus anthracis* | pXO1 | Bacilli | 181677 | Non-mobilizable | NZ_CP015777 |
| Syn_HAS | BACE001.B.00009.P002 | *Bacillus cereus* | p03BB102_179 | Bacilli | 179680 | Non-mobilizable | NC_012473 |
| Syn_HAS | BACE001.B.00010.P002 | *Bacillus cereus* | pCI-XO1 | Bacilli | 181907 | Non-mobilizable | NC_014331 |
| Syn_HAS | BACE001.B.00017.P002 | *Bacillus cereus* | NA | Bacilli | 179680 | Non-mobilizable | NZ_CP009317 |
| Syn_HAS | BACE001.B.00020.P003 | *Bacillus cereus* | pBCX01 | Bacilli | 190860 | Non-mobilizable | NZ_CP009592 |
