# Supplementary material for: Genetic exchanges are more frequent in bacteria encoding capsules
Source: PLoS Genet. 2018 Dec 21;14(12):e1007862. doi: 10.1371/journal.pgen.1007862 (PMC6322790; doi:10.1371/journal.pgen.1007862)
Supplement: S7 Table — (DOCX) [file pgen.1007862.s020.docx]

**Table S7. List of capsule systems found in prophages.**

| **Database ID** | **Prophage host** | **Capsule type** | **Prophage size** | **Position in genome** | **Accession** |
| --- | --- | --- | --- | --- | --- |
| SAEN001.B.00002.C001 Ph-05 | *Salmonella enterica* Typhi str. Ty2 | Group II (ABC) | 93168 | 4442986-4536153 | NC_004631 |
| SAEN001.B.00020.C001 Ph-06 | *Salmonella enterica* Typhi str. CT18 | Group II (ABC) | 93859 | 4459393-4553251 | NC_003198 |
| SAEN001.B.00041.C001 Ph-05 | *Salmonella enterica* Typhi str. Ty21a | Group II (ABC) | 93168 | 4442983-4536150 | NC_021176 |
| SAEN001.B.00143.C001 Ph-05 | *Salmonella enterica* Typhi | Group II (ABC) | 67647 | 4417047-4484693 | NZ_CP012151 |
| LAPL001.B.00004.C001 Ph-02 | *Lactobacillus plantarum* ZJ316 | Group I (Wzy) | 92763 | 2066704-2159466 | NC_020229 |
| LAPL001.B.00005.C001 Ph-01 | *Lactobacillus plantarum* P-8 | Group I (Wzy) | 101602 | 1682033-1783634 | NC_021224 |
| LAPL001.B.00008.C001 Ph-02 | *Lactobacillus plantarum* | Group I (Wzy) | 89659 | 1858303-1947961 | NZ_CP010528 |
| BATH001.B.00012.C001 Ph-04 | *Bacillus thuringiensis* YBT-1518 | Group I (Wzy) | 67839 | 1787270- 855108 | NC_022873 |
| BASE001.B.00001.C001 Ph-02 | *Bacillus selenitireducens* MLS10 | Group I (Wzy) | 97041 | 3193009-3290049 | NC_014219 |
