# Supplementary material for: Genetic exchanges are more frequent in bacteria encoding capsules
Source: PLoS Genet. 2018 Dec 21;14(12):e1007862. doi: 10.1371/journal.pgen.1007862 (PMC6322790; doi:10.1371/journal.pgen.1007862)
Supplement: S8 Table — A. Number of orthologous proteins between the prophages in Salmonella bearing a capsule operon. Threshold for orthologous genes was set to 80% similarity. B. Identity (lower triangle) was calculated for the 59 proteins common to all prophages for all pairwise comparison using needle (Needleman-Wunsch) from the EMBOSS package v6.6.0.0 with default options (-gapopen 10.0 -gapextend 0.5) using the proteic sequence. Weighted gene repertoire relatedness (wGRR, upper triangle) was calculated as ∑i=1MS(Ai,Bi)min(ηA,ηB), with S(Ai,Bi) representing the similarity score of the pair i of homologous proteins shared by phage A and phage B (bi-directional best hit), M the total number of homologs between phages A and B and ηA and ηB the total number of proteins of phage A and B, respectively. (DOCX) [file pgen.1007862.s021.docx]

**Table S8. Orthologous proteins and identity between the *Salmonella enterica* prophages. A.** Number of orthologous proteins between the prophages in *Salmonella* bearing a capsule operon. Threshold for orthologous genes was set to 80% similarity. B. Identity (lower triangle) was calculated for the 59 proteins common to all prophages for all pairwise comparison using *needle* (Needleman-Wunsch) from the EMBOSS package v6.6.0.0 with default options (-gapopen 10.0 -gapextend 0.5) using the proteic sequence. Weighted gene repertoire relatedness (wGRR, upper triangle) was calculated as $\sum_{i=1}^{M} \frac{S_{(Ai, Bi)}}{min(\eta_{A},\eta_{B})}$, with S_(Ai,Bi)_ representing the similarity score of the pair i of homologous proteins shared by phage A and phage B (bi-directional best hit), M the total number of homologs between phages A and B and $\eta_{A}$and $\eta_{B}$the total number of proteins of phage A and B, respectively.

Orthologous proteins

| **Database ID** | **Prophage host** | **# of proteins** | Typhi str. Ty2 | Typhi str. CT18 | Typhi str. Ty21a |
| --- | --- | --- | --- | --- | --- |
| SAEN001.B.00002.C001 Ph-05 | *Salmonella enterica* Typhi str. Ty2 | 105 |  |  |  |
| SAEN001.B.00020.C001 Ph-06 | *Salmonella enterica* Typhi str. CT18 | 80 | 77 |  |  |
| SAEN001.B.00041.C001 Ph-05 | *Salmonella enterica* Typhi str. Ty21a | 105 | 105 | 77 |  |
| SAEN001.B.00143.C001 Ph-05 | *Salmonella enterica* Typhi | 76 | 76 | 59 | 76 |

Identity & wGRR

| **Database ID** | **Prophage host** | Typhi str. Ty2 | Typhi str. CT18 | Typhi str. Ty21a | Typhi |
| --- | --- | --- | --- | --- | --- |
| SAEN001.B.00002.C001 Ph-05 | *Salmonella enterica* Typhi str. Ty2 |  | *0.961* | *0.99* | *0.998* |
| SAEN001.B.00020.C001 Ph-06 | *Salmonella enterica* Typhi str. CT18 | 99.9 |  | *0.961* | *0.775* |
| SAEN001.B.00041.C001 Ph-05 | *Salmonella enterica* Typhi str. Ty21a | 99.9 | 99.9 |  | *99.9* |
| SAEN001.B.00143.C001 Ph-05 | *Salmonella enterica* Typhi | 99.9 | 99.9 | 99 .9 |  |
